# Exploiting the CRISPR‐Cas9 gene‐editing system for human cancers and immunotherapy

**DOI:** 10.1002/cti2.1286

**Published:** 2021-06-22

**Authors:** Lukman O Afolabi, Mariam O Afolabi, Musbahu M Sani, Wahab O Okunowo, Dehong Yan, Liang Chen, Yaou Zhang, Xiaochun Wan

**Affiliations:** ^1^ Guangdong Immune Cell therapy Engineering and Technology research Center Center for Protein and Cell‐based Drugs Institute of Biomedicine and Biotechnology Shenzhen Institutes of Advanced Technology Chinese Academy of Sciences Shenzhen China; ^2^ University of Chinese Academy of Sciences Beijing China; ^3^ Department of Biochemistry Faculty of Science Federal University Dutse Dutse Nigeria; ^4^ Open FIESTA Center Tsinghua University Shenzhen China; ^5^ State Key Laboratory of Chemical Oncogenomics Graduate School at Shenzhen Tsinghua University Shenzhen China; ^6^ School of Life Sciences Tsinghua University Beijing China; ^7^ Department of Biochemistry College of Medicine University of Lagos Lagos Nigeria

**Keywords:** CRISPR‐Cas9, genetic manipulation, cancer, immunotherapy, T cells, natural killer cells

## Abstract

The discovery of clustered regularly interspaced short palindromic repeats and CRISPR‐associated protein 9 (CRISPR‐Cas9) technology has brought advances in the genetic manipulation of eukaryotic cells, which has revolutionised cancer research and treatment options. It is increasingly being used in cancer immunotherapy, including adoptive T and natural killer (NK) cell transfer, secretion of antibodies, cytokine stimulation and overcoming immune checkpoints. CRISPR‐Cas9 technology is used in autologous T cells and NK cells to express various innovative antigen designs and combinations of chimeric antigen receptors (CARs) targeted at specific antigens for haematological and solid tumors. Additionally, advanced engineering in immune cells to enhance their sensing circuits with sophisticated functionality is now possible. Intensive research on the CRISPR‐Cas9 system has provided scientists with the ability to overcome the hostile tumor microenvironment and generate more products for future clinical use, especially off‐the‐shelf, universal cellular products, bringing exciting milestones for immunotherapy. This review discussed the application and challenges of CRISPR technology in cancer research and immunotherapy, its advances and prospects for promoting new cell‐based therapeutic beyond immune oncology.

## Graphical abstract

This review discusses the CRISPR‐Cas9 gene‐editing system and how it can be exploited for cancer research and immunotherapy. We also highlight its challenges and prospects for the creation of new cell‐based non‐immuno‐oncology therapy in the future.

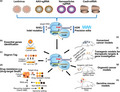

## Introduction

Despite concerted global efforts to control this disease, cancer continues to be a significant health burden, in spite of the advancements in treatment options such as radiotherapy, surgery, chemotherapy and, more recently, immunotherapy. Cancer is the world's second leading cause of death due to constant metastasis and relapse.^1^ Therefore, the fight against cancer is a global concern, which calls for new treatment strategies.

In the past, attempts to edit eukaryotic cells, particularly immune cells using the available genetic tools, have yielded little success. The ability of deoxyribonucleic acid (DNA) to repair itself after a double‐stranded break provides an avenue for genetic manipulation. The clustered regularly interspaced short palindromic repeats and CRISPR‐associated protein 9 (CRISPR‐Cas9) technology represents one of the high‐throughput gene‐editing technologies that have revolutionised available treatment options for many human diseases, including cancer.^2^, ^3^ CRISPR‐Cas9 offers a flexible and advanced gene‐editing capability compared with other gene‐editing technologies such as ribonucleic acid interference (RNAi), transcription activator‐like effector nucleases (TALENs) and zinc finger nucleases (ZFNs).^4^ Besides, CRISPR offers the potential to multiplex multiple gene targets, program its guide RNA (gRNA) and ease of *in vivo* delivery with low cytotoxicity.^5^ The CRISPR toolkit has been applied to multiplex genetic research with great success.^6^ Other research areas that have benefited from the CRISPR‐Cas9 system include neurological, skin and genetic disease therapies.^7^

Here, we describe the CRISPR‐Cas9 gene‐editing system and discuss how it has been exploited for cancer research and immunotherapy. We also highlight its challenges and prospects for the creation of new cell‐based non‐immuno‐oncology therapy in the future.

### The CRISPR–Cas9 biology and mechanism

The CRISPR‐Cas9 concept originated from the adaptive immune system of prokaryotes against foreign or invading DNA from bacteriophages.^8^, ^9^, ^10^ Prokaryotes (bacteria and archaea) acquire short genome segments (spacers) from the invading phage, which they integrate into their genetic code to serve as molecular memory during any subsequent infection by the same invading organism.^10^, ^11^ The acquired short sequence is then transcribed after maturation as part of the CRISPR array to form the CRISPR RNA (crRNA), which serves as a guide to the Cas9 endonuclease to scan and cleave any invading genetic material that matches the genetic target.^7^, ^12^ Cleavage of the genetic target is usually at the site that predates the protospacer adjacent motif (PAM). This biological defence system has been widely adapted for genomic engineering across various species from microbes, plants and animals.^7^, ^13^

### CRISPR‐Cas9 mechanism of action

The CRISPR‐Cas9 system can be regarded as an RNA‐guided endonuclease (RGEN), which involves recognising specific short target sequences (~20‐bp). The system employs a guide RNA to recognise its target nucleotide, followed by Cas9 nuclease activity.

In principle, the CRISPR‐CAS system works following two crucial steps:

- Sequence recognition (foreign nucleotide sequence)
- Nuclease cleavage (on identified target sequence), assisted by gRNA and Cas9 effector proteins.

The PAM, a 2–6‐base pair nucleotide sequence, is highly essential for the gRNA to recognise its target nucleotides (~20‐bp), followed by the recruitment of the Cas9 protein.^11^ The gRNA then guides the recruited Cas9 through its specific sequences related to a transactivating crRNA (tracrRNA) to form the complementary DNA target sequence for the site‐specific double‐strand break. Interestingly, CRISPR‐Cas9 can simultaneously cleave multiple genes,^14^ thus serving as an ideal tool for cancer research and the advancement of various therapeutic options, such as immunotherapy.

In endogenous systems, nuclear cleavage begins when mature CRISPR RNA (crRNA) fuses with transactivating crRNA (tracrRNA), which gives rise to a Cas9‐guided complex that leads to the target site of the invading DNA (protospacer).^9^ However, researchers have developed a gRNA as an artificial replacement for the endogenous crRNA complex.^15^

Ideally, DNA repair in the cell can occur via the non‐homologous end‐joining (NHEJ)‐mediated DNA pathway or by homology‐directed DNA repair (HDR).^16^ The former (NHEJ) involves direct ligation of the two single‐stranded ends, with resultant small random insertion or deletion mutations (indels)^17^ while the latter (HDR) requires a template donor DNA sequence with homologous arms to generate DNA repair,^18^ where programmed single‐strand DNA fragments are introduced to achieve insertion of a specific gene, also known as gene knock‐in. Lately, another repair mechanism known as microhomology‐mediated end joining (MMEJ) has been Identified.^19^, ^20^ It involves repairing DNA breaks through elongation from substantial microhomology arms (5‐ to 25‐bp sequences), usually generating indels.^21^ One unique advantage of the Cas proteins is that single‐ or dual‐guide RNAs can be designed and generated easily.

### Advantages of CRISPR over ZFNs and TALENs

The CRISPR system, when compared to other genetic tools such as ZFNs and TALENs, offers many advantages which include the following.

First is the design simplicity. Since the CRISPR system target recognition relies on forming a ribonucleotide complex rather than protein/DNA recognition, gRNA design is easier for any genomic targets.^2^ The second is its efficiency. The CRISPR system is highly efficient in terms of its actual genetic editing workflow; for example, mouse embryos can easily be modified by the direct delivery of RNAs encoding the Cas protein and its gRNA into them, thus eliminating the hurdles and difficulty associated with the classical homologous recombination techniques.^5^ The third is its multiplex potential. The CRISPR system offers the ability to modify several genomic sites simultaneously by injecting with multiple gRNAs, and this has been used to simultaneously introduce five different gene mutations in mouse ES cells.^22^ Recently, it is now easy to also predict its off‐target sites, thereby maximising its efficiency.^4^

### CRISPR‐Cas9 for cancer research and drug targets

Cancer remains a global burden, with an unprecedented annual death. Cancer is characterised by several point mutations leading to an altered genome, and DNA damage, resulting in abnormality in cell division. However, the CRISPR‐Cas9 system has shown immeasurable success for studying normal and aberrant genes in cancer cells in various mouse models. For example, by combining the Cre–LoxP technology with the CRISPR–Cas9 system, a phenotypic deletion of tumor suppressor genes such as p53 and PTen was induced. This deletion could be accomplished either individually or in combination using CRISPR's hydrodynamic injection of a designed DNA plasmid expressing Cas9 and targeting these genes in the liver.^23^ Another study involving adeno‐associated virus (AAV) delivery of CRISPR plasmid to model p53, KRAS and LKB1 genes in lung adenocarcinoma caused mutation in p53 and LKB1, resulting in loss of function in these genes, followed by the formation of adenocarcinoma pathology mediated by homology‐driven repair of KRAS G12D mutations.^24^

CRISPR‐Cas9 was used to assess putative and novel targets, including the functional roles of cancer‐associated mutations in the spliceosome genes. The Degron‐KI system consisting of CRISPR‐Cas9‐mediated knock‐in of inducible degron tags was used to determine the causal link between the splicing changes of the SF3B1 hotspot mutations.^25^

The CRISPR‐Cas9 has also provided unparalleled usefulness in mimicking structurally aberrant chromosomes, which were previously tricky to model. This approach is relatively easy for insertion or deletion of DNA fragments of varying sizes in the human and mouse genome by the NHEJ/HDR pathways of CRISPR. Likewise, CRISPR technology has made it possible to generate duplication and deletion of DNA fragments by trans‐allelic recombination, creating double‐strand breaks (DSB) induced by Cas9 on homologous chromosomes, providing a model for the study of millions of gene clusters as well as many regulatory DNA clusters.^26^

It is now possible through a virally assisted CRISPR‐Cas9 delivery system to specifically induce *in vivo* chromosomal rearrangement in somatic cells in animals. The generation of an echinoderm microtubule‐associated protein‐like 4 gene fused to the anaplastic lymphoma kinase gene (Eml4‐Alk), which drives lung cancer mouse model, expressing the Eml4‐Alk fusion gene, shows the typical molecular and histopathological features of the human ALK^+^ non‐small‐cell lung cancer (NSCLC)^27^; such an approach can be modelled to investigate other genes implicated in the aetiology of other cancer types.

Furthermore, the use of CRISPR‐Cas9 for investigating multiple gene targets has led to the synthesis and creation of a genetic circuit that can aid cancer cell identification with strict specificity and efficacy of cancer gene therapies.^3^ This circuit approach involves integrating two promoters as input in a cell, and the output gene is activated only upon the dual activation of the input genes; this has been established for genes such as p21, E‐cadherin and hBAX, which inhibited cell growth, cell motility and induced apoptosis as a result of its corresponding genes.^28^

Chemotherapy represents one of the most common cancer treatment options, and drug resistance is a stumbling block to the success of many therapies; therefore, the search for novel antineoplastic drugs has become imperative. The CRISPR system has been employed as part of the approach to predict and validate novel drug targets. One of such approach is the Drug Target SeqR, designed to find physiological drug targets, which involves the combination of computational mutation discovery, high‐throughput sequencing and genome editing mediated by the CRISPR‐Cas9. The process consists of inducing protein mutation, which confers drug resistance and reduces cell activity when tested in biochemical assays. An example of such a drug target discovered by this approach is ispinesib (kinesin‐5 inhibitor) – an anticancer inhibitor that causes cell death in actively dividing tumor cells.^29^

Another potential cancer drug (selinexor) target was identified and validated by the CRISPR‐Cas9 system. Selinexor is an exportin‐1 (XPO1) inhibitor, and the CRISPR‐Cas9 system was used to show that resistance of cancer cells to this drug was because of mutations at the cysteine‐528 in the XPO1 gene.^30^

**Figure 1:**
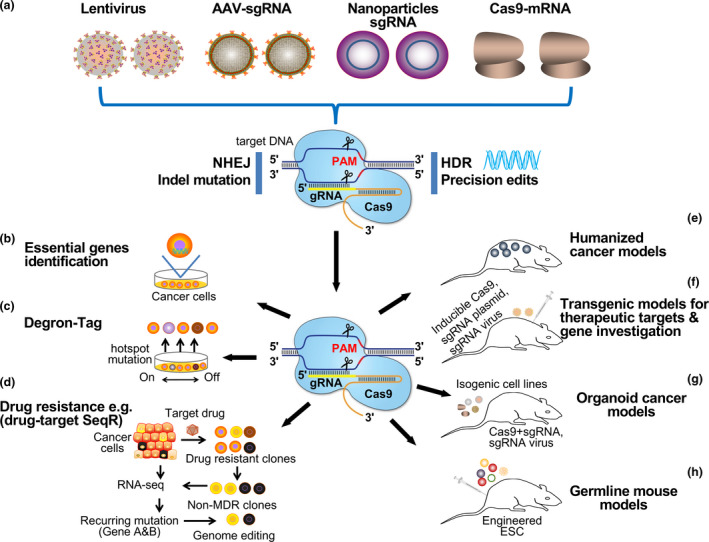
Application of the CRISPR‐Cas9 system in cancer research and therapeutics. **(a)** Various delivery methods of the CRISPR‐Cas9 material. They range from lentivirus, adeno‐associated virus (AAV), nanoparticles and Cas9‐mRNA. The CRISPR system can employ either the non‐homologous end joining (NHEJ) or the homology‐directed repair (HDR) for gene knockout and knock‐in, respectively. **(b)** The identification of essential genes or gene clusters peculiar to individual cancer cells. **(c)** Target validation mediated by degron tag knock‐in in a gene subjecting its expression to the presence of a small molecule. **(d)** Schematic workflow of DrugTargetSeqR application in identifying a drug's direct target gene curated from recurring gene mutation between parental cancer cells and non‐MDR clones, which can be validated by biochemical assays to ascertain whether mutations are sufficient to confer resistance. **(e)** CRISPR‐Cas9 mediated generation of humanised mouse strains carrying physiological levels of gene expression. Their endogenic gene expression levels make them essential components for human biology and pathology modelling, including the study of dosage‐sensitive genes such as aggregate sensitive proteins and RNA‐binding proteins **(f)** Cas9 mediated transgenic mouse models mediated by the delivery of viral sgRNA. Co‐expressing and/or inducible Cas9 enzymes can cause tissue‐specific gene knockout in different organs. **(g)** CRISPR‐Cas9 generation of mutation (point or compound) by chromosome translocation or deletion in different mouse tissues, generating a panel of isogenic cell lines with a variety of oncogenic lesions. **(h)** Generation of germline mouse models harbouring several genetic mutations mediated by CRISPR‐Cas9 engineered embryonic stem (EM) cells.

Besides drug target discoveries, other chemotherapy problems such as multidrug resistance against anticancer drugs are also challenges. The CRISPR‐Cas9 system can help identify the gene(s) responsible for drug resistance and test whether any single mutation in such gene(s) or knock‐in/out of target genes can confer drug resistance in different tumors. Such an approach will be convenient to reliably generate *in vitro* and *in vivo* models for thorough and high‐throughput basic research and preclinical investigation on candidate genes and elucidate the responses of cells in the presence or absence of such target gene(s) (Figure 1).

Another significant benefit of the CRISPR technique is identifying which proteins cancer cells depend on for survival, thus identifying other potential drug targets. This process involved the identification of functional protein‐coding exon, which could serve as new targets. For example, Shi *et al*. screened 192 regulatory chromatin domains in mouse acute myeloid leukaemia (AML) cells, 19 new drug targets and six known drug targets were identified.^31^ Similarly, the CRISPR‐Cas9 system targeted at the promoter of the human papillomavirus (HPV) (E6 and E7 transcript region) resulted in the accumulation of p21 and p53 proteins, leading to a reduction in the proliferation of cancerous cells (both *in vivo* and *in vitro*), thus demonstrating the usefulness of CRISPR for high risk‐HPV oncogenes and HPV‐related cancer treatment.^32^

CRISPR‐Cas9 studies targeting multiple genes may hold the key to treating multiple mutations involved in heterogeneous tumor mass in NSCLC. This system is a better alternative to lung cancer therapy involving histone deacetylase or DNA methyltransferase (DNMT) as it does not have many of the after‐effects of DNMT inhibitors.^25^ It also enables the target of epi‐enzymes to study the epigenetic modulation, control and expression status of cells by recruiting effector domains, including any major chromatin remodelling complexes.

It is also possible to construct a CRISPR‐Cas9‐based sequence to probe and identify novel regulatory gene clusters unique to specific cancer features. Based on this approach, a novel mutation that elicits resistance against the PLX‐4720 (a potent and selective inhibitor of BRAF^V600E^) in melanoma cells was identified.^33^ These genome screenings have a lot of potential because they allow for the identification of epigenetic marks within the cancer genome when combined with bioinformatics approaches. Other cancer therapeutic areas that CRISPR‐Cas9 can exploit for genetic transcripts include RNAs, antisense transcripts, polymerase III transcripts, non‐coding RNAs, nuclear‐localised RNAs, microRNAs, polymerase III transcripts with such large variety sequences that can be targeted, including promoters and introns.^34^ Employing this technology in genome and epigenome editing is expected to lead to numerous new treatment options in one of the deadliest human diseases.

### Exploiting genome‐wide CRISPR‐Cas9 screening for cancer therapeutic

The genome‐wide CRISPR‐Cas9 screen entails disrupting gene functionality with sgRNA to uncover novel yet unidentified targets and pathways that influence many biological processes.^35^, ^36^ Since its emergence, many studies have developed genome‐wide CRISPR knock‐out (GeCKO) libraries harbouring arrays of sgRNAs targeted towards a set of genes implicated in cancer aetiology. Since the designed sgRNAs alter and modulate the targeted gene’s role in cell viability during proliferation, the depletion or enrichment of these sgRNAs identifies the genes implicated in the observed cell phenotype^37^, ^38^, ^39^, ^40^, ^41^. The CRISPR genome‐wide screening was shown to identify a novel target in AML tumor cell lines. The knockout of the transcriptional activator KAT2A alters their growth. Although KAT2A is not an essential gene for hematopoietic progenitor cells, targeting this gene represents a novel strategy for AML treatment, including the use of MB‐3 – a potent inhibitor of KAT2A for AML treatment.^42^

Moving forward, the genes responsible for bortezomib (BTZ) resistance in multiple myeloma (MM) were uncovered via a genome‐scale positive selection assay involving culturing MM cells harbouring various sgRNA targets in the presence of a lethal BTZ dosage. PSMC6 was identified as conferring resistance to BTZ in this cell after surviving‐conferring genes were enriched in sgRNAs sequencing.^43^

Another exciting study employing sgRNAs targeted at 2368 murine genes unravelled the protein tyrosine phosphatase non‐receptor type 2 (Ptpn2) as a resistance‐conferring gene to programmed death ligand 1 (PD‐L1) blocking and its loss improves PD‐L1 immunotherapy.^44^ Another study showed that the loss of GRB2, IRF4, SOS1 and STAT3 in ALK^+^ anaplastic large‐cell lymphoma cells dampened PD‐L1 expression and restored T‐cell and NK‐cell antitumor functions.^45^

The identification of novel immunomodulatory compounds can be used to augment conventional chemotherapy care. For example, the mechanism of MM cell lines susceptibility to immunomodulatory imide drugs (IMiDs) was explored by loss‐of‐function genome‐wide screening and found that COP9 signalosome complex subunit 9 mediates the regulation of cereblon, which serves as the main factor responsible for sensitivity of MM cells to IMiDs.^46^

### CRISPR‐Cas9 genome screening for TCR and CAR‐T cells

The roles of cytotoxic T cells in the control of tumors have been well established. However, despite the advances in adoptive T‐cell immunotherapies and other novel T‐cell‐based therapeutics, malignant refractory and immune escape by some tumor cells remains a significant burden. In the past, gene knockdown attempts have been made using RNA interference libraries to identify targets that enhance T‐cell functions and understand how T cells respond when they encounter their target antigens.

CRISPR‐Cas9 ushered in a new gene perturbation approach known as CRISPR‐Cas9 genome‐scale screening. This functional genetic perturbation approach has been applied in many genetic studies, including primary T cells, to identify intrinsic T‐cell factors vital for an enhanced T‐cell cytotoxicity by employing an unbiased genetic screening approach.^47^

The CRISPR‐Cas9 genome screening involves generating a large pool of T cells (mediated by lentiviruses or other retroviruses encoding large libraries of perturbed genes) harbouring diverse edited genes traceable by their sgRNA sequences in the integrated CRISPR cassette. The CRISPR genome screen can then be coupled with single‐cell RNA sequencing (scRNA‐seq) to provide a powerful approach to evaluating each gene perturbation effect on the cell state and key signalling signature for its effector functions.

In principle, the CRISPR‐Cas9 genome screen is based on three components – (1) gene perturbation, (2) an applicable model and (3) an appropriate assay – to investigate the curated top hits genes.^47^

CRISPR genome screens in human T‐cell‐based therapies have been used to unravel target genes, including key signalling pathways that modulate the effector function of T cells. For instance, one way the Genome‐wide CRISPR screens have been used to enhance the effector function of CAR‐T cell is through a comprehensive study that identifies targets that can be translated to novel immunotherapies or an enhancement of existing therapy with gene‐engineering, biologics and small molecules.

Based on large‐scale CRISPR screens, a new method termed ‘SLICE’ was developed by Shifrut *et al*. to discover new regulators in primary human T cells that impacted its stimulation responses. This genome‐wide loss‐of‐function screen identified certain critical T‐cell‐positive genes – LCP2 and negative genes – CBLB, CD5– signatures as important for TCR signalling. Additionally, the authors identified genes resistance to adenosine‐mediated immunosuppression, which enhanced T‐cell proliferation in the presence of adenosine agonist (CGS‐21680) when the identified genes are knocked out.^48^ Evidently, the described approach will significantly improve TCR‐based T‐cell therapies.

About 10–20% of patients with acute lymphoblastic leukaemia (ALL) show resistance after CD19‐directed CART19 treatment without a clear understanding of the development of such resistance. Using the CRISPR screen approach, an inherent impaired death receptor signalling in ALL patients was identified to directly correlate to failed CAR‐T therapy through impairment of T‐cell cytotoxicity, ultimately resulting in CAR‐T cell dysfunction. This study demonstrates a novel antigen‐independent mechanism of resistance to CART19 therapy.^49^

In another closely related report, the use of CRISPR‐Cas9 loss‐of‐function screens with a systematic investigation of druggable mechanisms of CAR‐T cell cytotoxicity of over 500 small molecules revealed some tyrosine kinase inhibitors that transcriptionally impede T‐cell signalling, thereby impairing CAR‐T cell cytotoxicity. Interestingly, the identification of death receptor signalling mediated via the FADD and TNFRSF10B (TRAIL‐R2) signatures was also implicated as a key mediator of CAR‐T cell cytotoxicity, which further elucidate the RIPK1‐dependent mechanism of SMAC mimetic sensitisation of diffuse large B‐cell lymphoma cells and B‐cell acute lymphoblastic leukaemia to anti‐CD19 CAR T cells.^50^ Since death receptors have varied expression profiles across genetic subtypes of B‐cell malignancies, this highlights a direct link between the mechanistic cytotoxicity of CAR‐T cells and cancer genetics.

In another interesting study, using a reciprocal CRISPR screening approach, Wang *et al*. revealed genes in both CAR‐T and tumor cells regulating cytotoxicity of CAR‐T cells while identifying the target genes critical for patient‐derived cancer stem cells susceptibility to such CAR‐T‐mediated killing. In their study, they discovered a novel CAR‐T cell‐ and tumor‐intrinsic target that improved *in vitro* and *in vivo* cytotoxicity against Glioblastoma stem cells (GSCs). Genetic ablation of identified hits in CAR‐T cells enhanced the cytolytic activity, long‐term activation and improved *in vivo* antitumor cytotoxicity against GSCs. Similarly, the knockout of identified targets hits in GSCs sensitised them to *in vitro* and *in vivo* CAR‐mediated cytolysis.^51^ This reciprocal CRISPR screening can be used to design and find a potential combinatorial inhibitory treatment strategy that would augment CAR‐T cell tumor clearance efficacy and promote advanced immuno‐oncotherapy.

Besides the CRISPR‐Cas9 genome‐scale knockout approach, Roth *et al*. demonstrated a widely adaptable non‐viral DNA CRISPR‐Cas9 genome‐scale knock‐in screens in primary human T cells. In their approach, dozens of uniquely barcoded large non‐viral DNA templates construct were knocked‐in into the TCR locus to unravel the candidate constructs that enhanced the fitness and functionality of the engineered T cells both *in vitro* and *in vivo*. Their pooled knock‐in sequencing (PoKI‐seq) combined with single‐cell transcriptome analysis was used to identify a novel transforming growth factor b (TGF‐b) R2‐41BB chimeric receptor constructs that significantly improved solid tumor clearance.^52^ Such laudable pooled knock‐in screen approach will allow the gene knock‐in of a large multiplexed library of DNA constructs to endogenously modify genetic sequences to generate and accelerate the discovery of more effective T‐cell therapies.

The CRISPR‐Cas9 genome‐scale knockout offers the platform to knock out canonical checkpoint genes such as PD‐1 or other immune‐suppressive genes, followed by an extensive assay to identify critical elements/pathways responsible for such negative immune signals which could be targeted via gene ablation or pharmacologically. Additionally, CRISPR genome‐scale pooled knock‐in (such as PoKI‐seq) offers the ability to rewrite the endogenous genetic signatures of immune cells, particularly T cells, to improve tumor specificity and resistance to exhaustion, homing to the tumor site with augmented tumor cytotoxicity. Employing such an approach for adoptive TCR and CAR‐T‐cell therapies holds much promise in developing functional and clinically relevant T‐cell‐based therapies.

### CRISPR‐Cas9 in immunotherapy

The hallmark of failed cancer therapies is immune escape by tumor cells that circumvents the numerous antitumor immune responses. Hence, cancer immunotherapy seeks to understand the immune system's complexities in relation to cancer cells in order to harness and augment natural immune mechanisms to combat the disease. Simply put, cancer immunotherapy entails innovative treatment options, unlike traditional cancer treatments such as radiotherapy and chemotherapy. It offers an incomparable advantage with extended progression‐free survival and overall survival in patients. Its dynamic and innovative therapies entail reinvigorating the endogenous antitumor immunity against cancers via several directions.^53^ Therefore, immunotherapy seeks to fortify components of the immune systems and modulate the complexity of the hostile tumor microenvironment (TME) such that immune cells can target tumor cells with high specificity and penetrate tumor sites to exert their antitumoral functions.^54^ Immunotherapy has shown to be highly efficacious with tumor‐targeting specificity when combined with conventional treatment options or designed with multiple immune checkpoint blockades (ICB). To achieve this, it is imperative to modify cytotoxic lymphocytes such as T and NK cells that are not easy to manipulate, considering the available genetic editing methods. The CRISPR‐Cas9 gene‐editing system provides a viable and safe alternative to generate clinically safe engineered T and NK cells for cancer immunotherapy.

### CRISPR‐Cas9 in chimeric antigen receptor (CAR) immunotherapy

The emergence of chimeric antigen receptor T‐cell (CAR‐T) therapy as a promising treatment option for cancer, particularly for haematological malignancies, is laudable.^55^ Engineered CAR‐T cells can be activated, infiltrate tumor sites, secrete cytokine and licensed to kill tumors in a manner that ensures complete tumor regression. Since CARs are usually designed for a specific tumor‐associated antigen, they consist of one or all of the following: an extracellular antigen binding domain, a hinge domain, a transmembrane region and an intracellular signalling domain.

**Figure 2:**
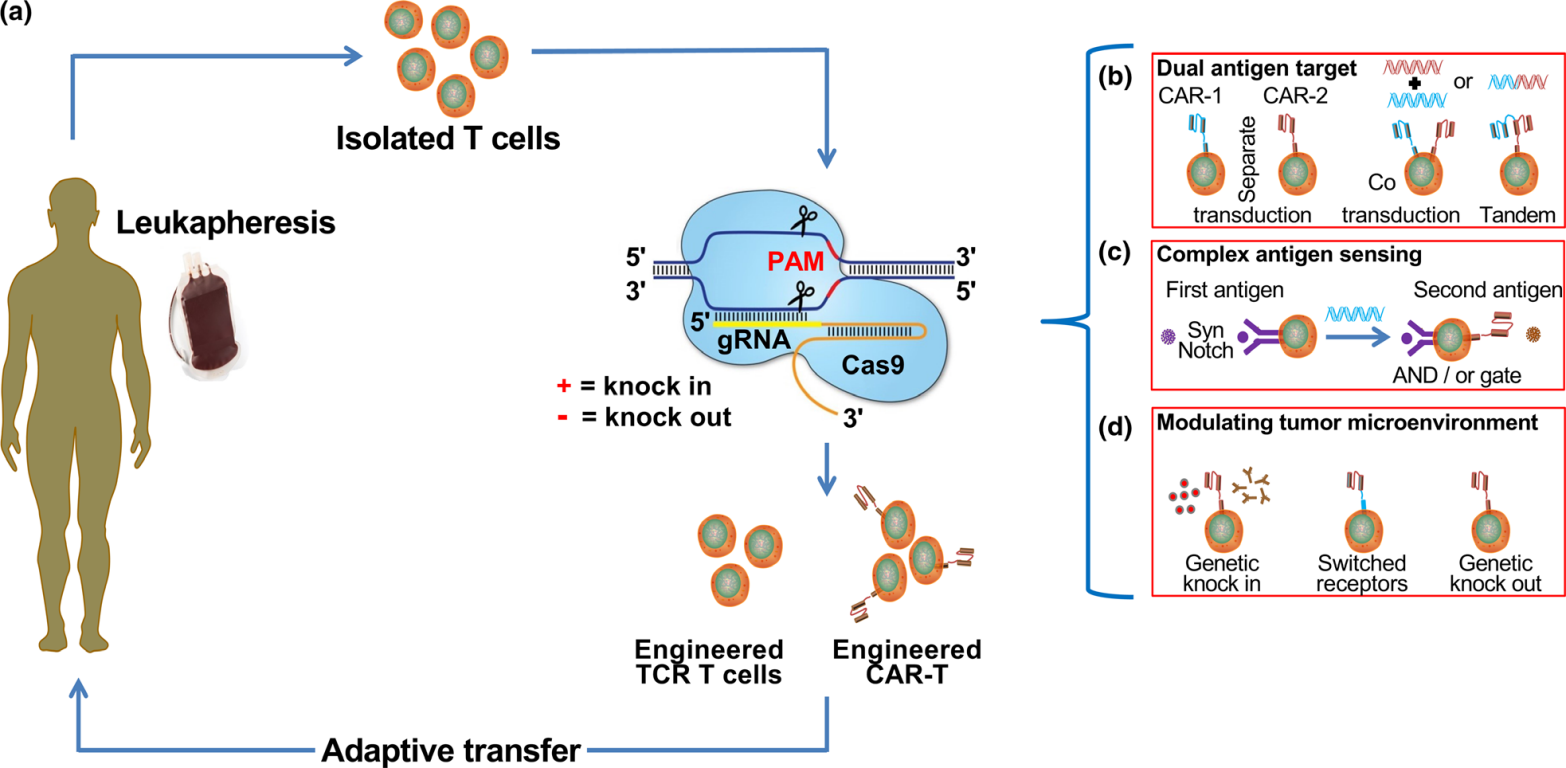
CRISPR‐Cas9 genome‐editing strategies in adoptive T‐cell immunotherapy for cancer. Applications of the CRISPR‐Cas9 in T‐cell cancer immunotherapy. **(a)** Isolated patient‐derived T cells are genetically engineered with CRISPR‐Cas9 to knockout endogenous genes, for example PD‐1, and knock‐in therapeutic TCR, and CARs, followed by *ex vivo* expansion and adoptive transfer. **(b)** CRISPR‐Cas9 inspired dual‐specific tumor recognition to overcome tumor heterogeneity or antigen loss. This can be achieved by transducing a single CAR molecule into two T‐cell populations (separate transduction), incorporating two CAR molecules into a single‐cell population either individually or by bicistronic (co‐transduction) and linking two separate CAR molecules to produce a single signalling chain (tandem transduction). **(c)** To surmount the off‐target effect and fine‐tune antigen sensing of tumor‐specific T cells, incorporating a synNotch receptor specific for a first antigen that can trigger the production of CAR upon interaction with a second antigen – this triggers its activation with a licence to kill the tumor. **(d)** Genetically reprogrammed T cells to overcome the hostile tumor microenvironment. The incorporation of genes capable of local cytokines or antibody release. Similarly, switched receptor strategies enhance sustained antitumor response and the deletion of inhibitory molecules or immune checkpoints to generate off‐the‐shelf T‐cell therapies.

Interestingly, most current CAR‐T cell clinical trials utilise autologous T cells from the patient’s own peripheral blood mononuclear cells. Although this is ineffective, attempts have been made to create a universal CAR‐T cell.^56^, ^57^ The CRISPR‐Cas9 system offers many alternatives to enhance the current CAR‐T and facilitates efficient and straightforward multiplex genomic modification of T cells to enhance its activation, tumor specificity and infiltration to improve the overall efficacy and safety of CAR‐T cells (Figure 2).

### Engineering CAR‐T cells with CRISPR‐Cas9

The therapeutic efficacy of CAR‐T cell has been shown especially for B‐cell lymphoma and other malignancies.^58^, ^59^ Currently, the standard CAR‐T treatment procedure required the autologous transfer of cells, which are often detailed, expensive and sometimes challenging to obtain sufficient qualitative T cells, especially in neonates and elderly, to generate patient‐specific CAR‐T cells.^60^

CRISPR‐Cas9 offers the potential to develop a universal CAR‐T (obtained from healthy donors) for allogeneic transfer, which has many advantages over autologous CAR‐T. The success of such an approach will be to delete the human leukocyte antigens class I (HLA‐Is) and subunits of the T‐cell receptor (αβ) – (*TRA* and *TRB*) on the allogenic CAR‐T cells.^60^ Mutation in the T‐cell receptor (TCRα) subunit constant (TRAC gene) of the T cell can lead to loss of its surface αβ TCR^61^; similarly, a mutation in the beta‐2 microglobulin (B2M) gene led to the loss of expression of HLA‐I heterodimers on the T‐cell surface ^62^. The generated B2M^–/–^embryonic stem cells (ESCs) could serve as universal donor cells where the transplanted cells do not express HLA class II genes.^62^

In another modified approach, Liu *et al*. showed that two (B2M and TRAC) and three (PD‐1, B2M and TRAC) genes could be effectively disrupted by the CRISPR technique to generate universal CAR‐T cells. By designing two sgRNAs each specific for the first exon of B2M and PD‐1, and another for the TRAC gene. The in vitro antitumor function of these multiplex double‐knockout (DKO) (TRAC and B2M) and triple‐knockout (TKO) (TRAC, B2M, and PD‐1) CAR‐T cells revealed higher cytokine production and potent cytotoxic activity against tumor cells compared to standard CAR‐T cells.^60^

Using a xenograft lymphoma mouse model, similar results were obtained for the *in vivo* effector function of these CAR‐T cells where a DKO and TKO was induced, leading to a significant reduction in tumor size, indicating that the CRISPR‐mediated multiplex gene deletion of HLA‐1 and TCR from CAR‐T cells retained their CD19‐specific antitumor function.^60^

In a closely related report, CRISPR‐Cas9‐mediated allogeneic CAR‐T cells show multiplex gene editing, the authors combined CAR lentivirus delivery with CRISPR RNA electroporation for co‐introduction of gRNA (specific for B2M, TCR and PD1 deletion). This approach describes the concept of engineering CAR‐T cells devoid of the TCR, programmed death protein (PD1 – immune checkpoint) and the HLA class 1 molecule, with potent *in vitro* and *in vivo* antitumor activity, compared to the unmodified CAR‐T cells. The DKO CAR‐T cell showed significantly reduced alloreactivity and did not elicit graft versus host diseases.^63^

Other promising studies include a CRISPR‐Cas9‐mediated CD19‐specific T‐cell targeting the α‐TCR subunit constant (TRAC); the method employed in this study resulted in the uniform expression of the CD19‐specific CAR on human peripheral blood‐derived T cells.^64^ By targeting the first TRAC exon, the gRNA and a repair matrix of AAV harbouring a self‐cleaving P2A peptide followed by cDNA of CAR were electroporated together with the Cas9‐mRNA to generate the engineered TRAC‐CAR‐T cell. The efficiency of these engineered CAR‐T cells (with TCR knockout) could be compared to other sequence‐specific strategies often employed to target different loci (CCR5, AAVS1, CD40L).^65^, ^66^, ^67^ Finally, the engineering of CAR‐T cells should use endogenous regulatory elements such as TRAC to avoid tonic signals, T‐cell exhaustion and delayed T‐cell differentiation while the CAR molecule can be re‐expressed after repeated antigen exposure.

Based on the above reports, it is evident that the generation of CAR‐T cells on a custom‐made patient basis is not sustainable. Such autologous T‐cell production remains the bottleneck for the large‐scale clinical application of CAR‐T therapies, considering the invested resources, cost and time. However, the inherent production failure associated with autologous T‐cell production, together with its restricted application on different cancer types, is enough to push for the development of universal ‘off‐the‐shelf’ CAR‐T cell therapies (Table 1), whose production and potential technical hurdles will be readily alleviated through the flexibility of the CRISPR system. This technique will improve the current CAR therapeutics while generating universal, programmable and flexible CAR‐T cells whose therapeutic effects are controllable. Embarking on such an approach will bring a paradigm shift in engineered universal CAR‐T that can be directly infused in recipients without re‐editing, albeit with multiple antigen target capabilities.

**Table 1:** The advantages of generating universal CAR‐T versus autologous CAR‐T

CAR‐T types	Cost of production	Time of production	Quality control	Availability
Autologous	Very high with complex logistics	Long time, even longer in neonates and elderly	Difficulty in controlling parameters in the production process because of variable starting cell population	Difficulty in obtaining qualitative starting patient’s cells could impact its production leading to failure to receive treatment
Universal	Relatively cheaper considering the number of recipients	Can be made in advance, with shorter, optimised production time, and made available to recipients on demand	Advanced production allows multiple rounds of quality control checks to ensure the product meets safety standard and quality	Stocks of pre‐manufactured CAR‐T products can be stored in a universal bank (similar to blood banks) and made available to recipients as when due

### Engineering TCR T cells with CRISPR‐Cas9

The CRISPR‐Cas9 system's efficacy in generating CAR‐based therapies targeted for CD19^+^ haematological malignancies cannot be overemphasised. It also plays a role in constructing TCR T cells through its multiplex approach to generate efficient T cells. In terms of surface antigen, presentations of major histocompatibility complex (MHC) independent, CAR‐based therapies have been used successfully against relevant tumors; however, engineered TCR T cells can identify tumor cells via the MHC complex, the antigenic peptides present on their surface. Interestingly, they do this via the antigenic peptide fragment/ MHC combinations. According to a report, TCR T cells can infiltrate solid tumors more effectively than CAR‐T cells.^68^

Studies have shown that tumor‐specific TCRs targeting the intracellular proteome and/or metabolome can be generated.^69^ Although some areas of concern have been identified, such as TCR mispairing – a condition of incorrect endogenous and recombinant TCR pairing, often resulting in reduced surface expression of therapeutic TCRs or sometimes autoreactivity.^70^, ^71^

The use of endogenous rather than engineered TCRs has been suggested; however, one of the major pitfalls of such an approach is the low‐affinity range of endogenous TCRs compared to engineered TCRs when targeting foreign pathogens, as most TAAs are self‐derived.^72^ Hence, therapeutic use of endogenous TCRs for cancer treatment can reduce efficacy with severe toxicity as these antigens also exist in normal cells. Despite the uncertainties and unintended consequences associated with the use of TCR T‐cell, the use of CRISPR‐Cas9 editing technique to induce endogenous knockout of TCRs has led to an increased surface expression of therapeutic TCRs, ultimately with improved sensitivity, specificity and cytotoxicity.^69^

Recently, a phase I trial, involving the transplantation of autologous T cells devoid of both endogenous TCR and PD‐1, was shown to improve their biosafety.^73^ Using the CRISPR‐Cas9 system for genome editing of autologous T cells by knockout of specific genes has helped researchers and clinicians explore the optimal therapeutic conditions for engineered TCR T cells. The goal of such engineered T cells is to enhance their functions while reducing the risk of autoimmunity.^73^ To this end, the CRISPR technique holds enormous possibilities for developing the next‐level TCR T cells for immunotherapy and beyond. Interestingly, the CRISPR‐Cas9 technology provides the avenue to do more basic research on TCR T cells to generate safe and better cell‐based products for clinical use, accelerating bench to bedside treatment.

### Strategies to augment natural killer (NK) cell antitumor activity and mitigate its exhaustion with CRISPR‐Cas9

The immune system plays a critical function in preventing the onset and metastasis of cancer. In this regard, NK cells represent an essential effector lymphocyte of the innate immune cells, and their antitumor roles have been well recognised.^74^, ^75^, ^76^ However, during tumor progression, NK cells are sometimes found exhausted within the TME. Numerous reports have demonstrated how the exhaustion of effector lymphocytes regulates and shapes the immune response to tumor progression and infections, limiting their antitumor potentials.

Since therapies targeted at activating and reinvigorating the immune effector functions can yield beneficial responses in patients with episodes of metastatic malignancies, this has led to long‐lasting clinical responses, thus revolutionising oncology with dramatic benefits in both haematologic and solid tumors. Based on the success recorded for reinvigorating exhausted T cells and enhancing their antitumor functions, extending this approach beyond T‐cell therapies is pertinent. Despite the documented success for T‐cell therapies, a critical assessment of the tumors originating from patients who progress on anti‐PD‐1 blockade showed an impaired antigen presentation and interferon signalling, leading to tumor evasion from T‐cell response. Unlike T cells, NK cells can exert their cytotoxicity on tumor cells without prior sensitisation to antigens, particularly tumor cells with low or impaired antigen presentation machinery.^74^ This makes approaches targeted towards preventing exhaustion of NK cells and reinvigorating their effector functions a laudable approach.

A critical understanding of the multiple mechanisms that might contribute to the anergy, exhaustion and senescence of NK cells, such as the presence of suppressive cytokines or soluble factors, regulatory immune cells and dysregulated receptor signals found within the TME, will guide to design modalities to augment NK‐cell functions. Besides creating novel NK‐cell‐based antitumor therapies, a clear understanding of the above characteristics will enhance our knowledge of basic NK‐cell biology and help overcome several hurdles limiting the clinical application of meaningful NK‐cell‐based therapies.

A review of the recent developments using the CRISPR system to augment NK‐cell effector function against tumors regarding NK‐cell immune checkpoints, cytokine therapy, NK‐cell engagers and adoptive infusion of NK cells is discussed below.

### Innovative NK cells engineering with CRISPR‐Cas9

Natural killer (NK) cells represent one of the first lines of the host immune surveillance. They play vital antiviral and antitumor roles on stressed or transformed cells through numerous mechanisms (e.g. direct cytotoxicity, secretion of cytokines/chemokines and antibody‐dependent cell‐mediated cytotoxicity). Unlike T cells, NK cells lack antigen‐specific recognition capability but play critical antitumor immunity roles.^77^ The use of NK‐cell immunotherapy is fascinating and represents a promising and dynamic strategy for cancer treatment, the antitumor effects of which require further improvement. In the past, attempts such as the use of antibodies, cytokines or gene‐editing have been embarked upon to overcome tumor immune suppression and enhance tumor recognition in NK cell immunotherapy.^78^, ^79^ CRISPR‐Cas9 genome‐editing system offers flexibility in editing NK cells *ex vivo* for adoptive therapy. Alternatively, this technique allows tumors to be manipulated *in situ* to increase their susceptibility to *in vivo* NK surveillance.^68^, ^80^

Recently, NK‐cell cancer immunotherapy has been explored for hematopoietic malignancies. Like the CAR‐T immunotherapy, CAR‐engineered NK cells have shown tumor target specificity and cytotoxicity.^81^, ^82^ The current preclinical and clinical applications and research on engineered CAR‐NK‐cell‐based immunotherapy targeted for different cancer types have been discussed.^68^, ^83^, ^84^ The immunotherapeutic effect of the diverse engineered CAR molecules on NK cells to redirect the corresponding specific antigens in a cell‐based approach has also been well discussed.^58^, ^84^, ^85^

**Figure 3:**
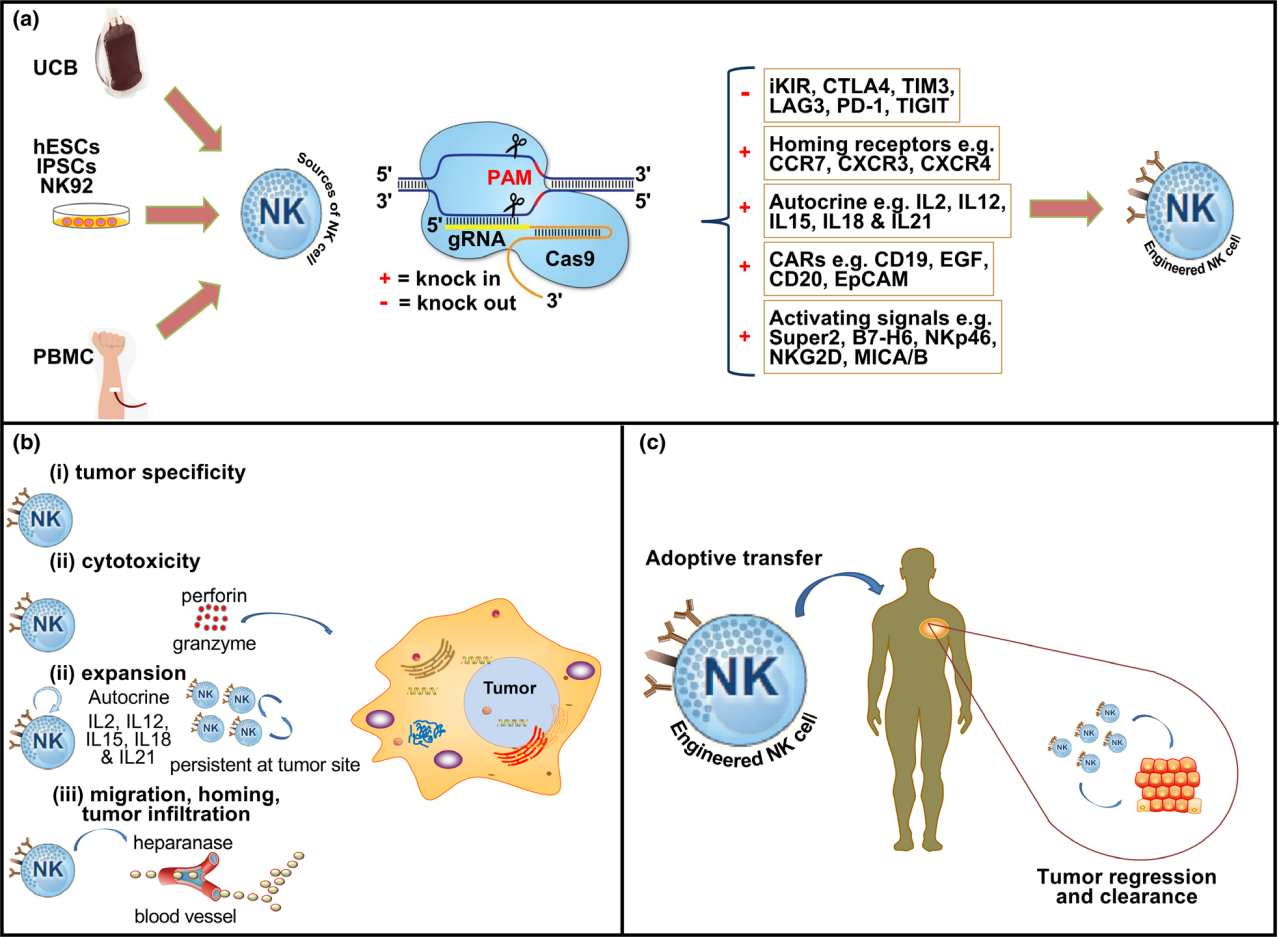
Overview of CRISPR‐Cas9 genome‐editing strategies for NK cell immunotherapy. **(a)** NK cell sources (UCB, umbilical cord blood; hESCs, hematopoietic embryonic stem cells; iPSCs, induced pluripotent stem cells; NK‐92, NK‐92 cell line; PBNK, peripheral blood mononuclear cells) and its manipulation via the multiplex capability of the CRISPR system. **(b)** Engineered NK cells with augmented antitumor capabilities such as tumor specificity, cytotoxicity, expansion and tumor infiltration. **(c)** Engineered NK cells adoptively transferred to confer tumor regression and clearance.

The NK cell is a potent effector cell, and its use in CAR targeted immunotherapy has numerous advantages compared to the T cell. For example, allogeneic NK cells kill target cells antigen‐independently, so they can be used for universal adoptive transfer, as they do not give rise to graft versus host diseases commonly seen in allogeneic T cells (HLA matching). Also, the inability of the CAR‐NK cells to induce cytokine storm also makes them safer than CAR‐T cells, and, finally, the abundance of sources for generating NK cells such as human peripheral blood (PBMC), umbilical cord blood (UCB), induced pluripotent stem cells (iPSCs), human embryonic stem cells (hESCs) and NK‐92 cell lines helps overcome the trouble of obtaining the cells in abundance^58^ (Figure 3).

Combining CRISPR‐Cas9 with another gene‐editing approach, Velasquez *et al*. reported a CAR‐NK‐based therapy bispecific T‐cell engager (CD19‐ENG) capable of targeting CD22^+^ B cells leukaemia as well as also redirecting T cells to kill malignant CD19^+^ B cells, hence preventing any immune escape by the tumor and improving its antitumor activity. For the first time, this study showed engineered CAR‐NK cells specific for CD22 and augmented CD19 T cell targeting of B‐cell malignancies.^86^ Such combined cytolytic target killing of malignant cells opens a new window in gene editing of cancer immunotherapy with a significant improvement in current B‐cell cell therapy and related malignancies.

These findings emphasise the enormous potentials of the CRISPR‐Cas9‐mediated gene editing of effector cells for clinical immunotherapies. Considering the strides already achieved in effector cell‐mediated immunotherapy, CRISPR‐based genetic manipulation has equipped scientists and clinicians with the new treatment tool that can be used to win the battle against many cancer malignancies. To attain such a feat, specific improvements need to be made. First, in the CAR‐NK design, the CAR molecules' introduction should be accomplished with the deletion of NK‐cell inhibitory receptors such as NKG2A or TIM‐3; this will confer sustained and intense cytotoxicity because of the lack of inhibitory signals usually encountered in the TME. Similarly, a multiplex TKO or DKO of inhibitory genes in NK cells as shown for CAR‐T cell (TCR, HLA and PD‐1/CTLA‐4/PD‐L1) should be given great attention.

A novel approach was suggested to overcome the immunosuppressive IL‐4 cytokine, which involves the inversion of the cytokine receptor (ICR) by fusion of the IL‐4 receptor exodomain with the IL‐7 receptor endodomain to generate a 4/7 ICR that confer IL‐4 immunosuppressive resistance to the CAR‐T cell while improving its cytotoxicity.^87^ Such an approach can be extended to engineered CAR‐NK cells with varying potential ICR endodomain candidates (IL‐15, IL‐18 and IL‐21) that still need to be fully established.

The targeted integration of the CAR genes at specific sites of the genome of effector cells is desirable compared to integration at a random site. The knock‐in of CAR at the α constant locus of TCR improved T‐cell antitumor activity.^64^ Similarly, the integration of CAR into the TRAC locus prevented CAR signalling and immune cell exhaustion. These approaches can be employed by the CRISPR‐Cas9 technique to generate CAR‐NK cells with improved antitumor efficacy.

Furthermore, the use of small inhibitory molecules such as BX795 (which inhibits TBK1/IKK complex by acting downstream of RIG‐I‐like receptor and TCR) to enhance CRISPR‐Cas9 material viral delivery can be explored^88^; this and other related non‐toxic molecules can significantly improve the genetic editing of these effector cells (T and NK cells) for immunotherapy. As previously stated, NK cells are potent effector cells with natural cytolytic, antiviral and antitumor functions. The preferred choice of NK cells as alternative immunotherapy is partly because of their lack of TCRs that could cause graft versus host disease, potentially generating off‐the‐shelf cell therapy. Although NK cells have an effector potential, they are sometimes dysfunctional in the TME.^89^ To this end, the CRISPR‐Cas9 system allows for genetic modification of NK cells to reinvigorate their cytotoxic, antiviral and antitumor immunity through the following means.

### Optimised innovative CAR molecules

The CRISPR‐Cas9 system allows NK cells to be fortified with CARs that target various tumor antigens.^90^, ^91^, ^92^ Loss of original tumor antigen is a concern for CAR‐based immune cell therapy. NK cells can be armed with pan‐specific CAR molecules to improve tumor recognition via multiple ligands, and hence elicit a superior antitumor response compared to a single ligand target. As proof of principle, NKG2D ligands, including (MHC class I chain‐related protein A (MICA) and B (MICB), and human cytomegalovirus UL16‐binding proteins, are poorly expressed in normal cells but highly expressed in virally transformed and tumor cells.^93^ Incorporating full NKG2D protein on T or NK cells as part of the CAR design with the potential of multiple tumor ligand recognition showed an enhanced antitumor effect against NKG2D ligand‐positive tumors.^94^, ^95^ Such pan‐specific CAR‐T or NK cells can also target NKG2D ligand‐positive myeloid‐derived suppressor cells (MDSCs) and regulatory T cells (Tregs), hence overcoming the immunosuppressive TME.^94^

To achieve full activation of CAR‐NK, the design of its intracellular domain should be different from CAR‐T. Since DAP12 has been shown to play a predominant role in the transduction of activating signalling in NK cells,^96^, ^97^ it is crucial to optimise the intracellular domains with special consideration for DAP12 combination to enhance the cytotoxic signals for CAR‐NK.

### Stimulating activating pathways

NK‐cell effector functions could be enhanced and sustained by activating receptors and cytokines (e.g. IL‐2, IL‐15, IL‐18 and IL‐21).^79^ IL‐2 and 15 have been established as essential for promoting NK‐cell survival.^98^ Additionally, IL‐2 mutant form ‘Super‐2’ reverses NK‐cell exhaustion and promotes its proliferation.^99^ The multiplex capability of the CRISPR‐Cas system can be used to force express one or more cytokines such as ‘Super‐2’, IL‐15 or other cytokines in enhancing NK‐cell survival and effector functions. The augmentation of the *in situ* expression of tumor‐specific ligands for activating NK cell receptors is another laudable approach.^100^, ^101^ It can enhance NK cell antitumor responses via activating pathways made possible by the CRISPR‐Cas9 system. For example, transcriptional activation of NKG2D ligands – MICA – has been done successfully using the CRISPR‐Cas9 method.^102^

### Enhancing NK cell infiltration

The homing and migratory ability of NK cells to the disease site, as well as its ability to infiltrate tumor tissues, is usually indicative of its success and good prognosis upon adoptive infusion during NK‐cell immunotherapy.^103^, ^104^, ^105^

The surface expression of specific chemokine receptors on NK‐cell‐targeted towards tumor‐specific ligands using the CRISPR‐Cas9 technology holds much promise. The therapeutic benefits of the engineered chemokine receptor – CXCR2 on NK cells – have shown enhanced migratory potential towards a chemokine gradient CXCR2 ligands,^106^ indicative of the enhancement of intratumoral infiltration of NK cells. Additionally, another separate report showed the increased migratory ability of NK cells genetically engineered with the chemokine receptor CCR7 towards its ligands (CCL‐19 and CCL‐21), offering tumor infiltration and homing.^107^, ^108^

Since the TME is a mosaic of different components, including the stroma, thorough profiling and optimisation of chemokine receptors required for maximum tumor penetration will be required to overcome tumor‐associated stroma impedance. To this end, engineered NK cells expressing the enzyme heparanase and other modifications such as CAR expression hold the propensity to improve NK‐cell tumor infiltration through the ability to degrade the extracellular matrix as this has been shown to be successful for CAR‐T cells,^109^ thus it can significantly improve NK‐cell immunotherapy.

### Overcoming NK cell inhibitory pathways

NK‐cell activation involves a balance between activating and inhibitory signals on its surface.^110^ Strikingly, tumor cells express ligands that prevent unwanted NK‐cell activation as part of their immune escape mechanism.^100^, ^101^ Inimical signals from checkpoint receptors are implicated in causing tumoral NK‐cell exhaustion.^89^ Besides, several reports have shown that the blockade of checkpoint receptors related to NK cells (such as CD96, NKG2A, PD‐1 or TIGIT) significantly improved its antitumor immunity.^111^, ^112^, ^113^ There is a paucity of information on the role of LAG3 on NK cells. Recently, LAG3 has been implicated to play an inhibitory role and is expressed by activated NK cells.^114^ Reports have demonstrated that the inhibitory signals received from LAG3 attenuate NK cell cytotoxicity, cytokine/chemokine release and its antitumor function.^114^, ^115^ Therefore, using the CRISPR‐Cas9 system to genetically disrupt pathways associated with some of the checkpoint cell‐surface receptors on NK cells might improve its effector functions.

### CRISPR‐Cas9 technology to improve immune checkpoint blockade

The increasing numbers of failed therapies targeted at cancer have brought about many novel cancer treatment strategies. In particular, ICB is one of the most successful cancer treatment options. The approach was pioneered with the application of monoclonal ICB antibodies: anti‐PD‐1/PDL1 antibodies and anti‐CTLA‐4. This was followed with drugs that explicitly target PD‐L1, for example atezolizumab, durvalumab and avelumab; and despite their initial promise, unintended cytotoxicity and some clinical failures raised significant concerns.^116^, ^117^, ^118^ One of the many ways to overcome this setback is to carefully elucidate the intrinsic expression of PD‐L1 by cancer cells – which has been implicated as the most immune evasion mechanism.^119^ Besides, since tumor expression of PD‐L1 has been correlated to the efficacy of immune checkpoint inhibitors across different cancer types.^120^ Therefore, it is logical and imperative to identify the mechanisms that regulate PD‐L1 expression to augment existing treatment options to aid the development of novel strategies. To this end, the CRISPR system can be employed. As a proof of concept, genome‐wide CRISPR screening has been used to identify an uncharacterised protein CKLF‐like MARVEL transmembrane domain‐containing protein 6 (CMTM6), which serves as a critical regulator for the surface expression of PD‐L1 – whose increased expression also correlated with enhanced tumor cell clearance with ICBs.^121^ Another closely similar CRISPR genome screening approach was employed to identify regulators of PD‐L1 expression in H358 lung adenocarcinoma; the authors identified SMAD4 and uroporphyrinogen decarboxylase (UROD) in addition to CMTM6 as novel regulators of PD‐L1 expression.^122^ Another report showed using the CRISPR‐based genome screening technique to identify another PD‐L1 regulator in human lung cancer cells. A role of the translation initiation factor EIF5B was identified in lung adenocarcinomas, whose overexpression, however, correlates with poor prognosis and is sufficient to induce PD‐L1.^122^

Palmer *et al*. used a CRISPR‐based method to knock out the cytokine inducible SH2‐containing (CISH) gene. In turn, CISH KO resulted in increased T‐cell receptor (TCR) avidity, tumor cytolysis and neoantigen recognition. However, the CISH KO led to increased PD1 expression, whose adoptive transfer synergises with PD1 blockade, with durable tumor regression and survival benefits in the preclinical animal model. This research identified a new avenue that modulates the recognition of neoantigens and the expression of their activation/exhaustion markers that dictate the functionality in tumor‐specific T cells.^123^

These findings and other similar CRISPR‐based approaches can be employed to elucidate mechanisms governing the immune checkpoint regulation and identify novel therapeutic targets for improved immunotherapy. Besides the above described, the CRISPR genome screens offer many advantages that when it is applied *in vivo*, for example, it is possible to model the complex interaction and replicate the dynamic TME. Therefore, *in vivo* CRISPR‐Cas9 genome screens now identify regulators of immune evasion by cancer cells, including immune cell inhibitors.^124^

However, the *in vivo* CRISPR genome screening is somewhat similar to *in vitro* approaches in which sgRNA is used to modify and generate mutant tumor cells, which are then transplanted via different routes and allowed to develop. Harvested tumors are then compared with unmodified tumors from immune‐competent mice to find any genetic hits that may play a role in the antitumor response.^124^

Several other studies have identified genes that could be targeted to promote tumor immunotherapy; for example, the loss of Ptpn2 and Adar1 was found to improve antigen presentation and tumor sensitisation to anti‐PD‐1 blockade to improve immunotherapy, respectively.^44^, ^125^ In a recent study, a novel CRISPR‐Cas9 system was used to knock out the cyclin‐dependent kinase 5 gene (CDK5), leading to the downregulation of PD‐L1 expression on tumor cells while promoting the population of cytotoxic effector cells in the TME.^126^

The role of epigenetic modifiers in antitumor immune response has been well identified^127^, ^128^, ^129^; CRISPR genome screen using epigenetic sgRNA has identified genes that confer the efficacy of anti‐PD‐1 blockade.^130^ Additionally, the histone chaperone Asf1a was reported to sensitise Kras/p53 tumor cells to anti‐PD‐1 therapy; and the loss of Asf1a also induced an inflammatory response, secretion of the cytokine – GMCSF, which modulates the polarisation of M1 macrophage and T‐cell activation.^131^ These reports reveal how the CRISPR system has been exploited to elucidate the various molecular mechanisms that govern the immune evasion of tumor cells. It is evident that CRISPR offers tremendous usefulness to identifying novel targets which may be explored to improve immune checkpoint therapy, particularly to overcome the recurrent resistance to immunotherapy.

## The FUTURE PROSPECTS for CRISPR‐Cas9

The CRISPR‐based technology has shown enormous potential in its routine clinical applications. Unlike the other gene‐editing tools, CRISPR offers many advantages, particularly in terms of its ease of *in vivo* delivery and the design of novel therapies for cancers.

### Current challenges and future perspectives for CRISPR technology in immunotherapy

One of the main concerns for the widespread use of CRISPR technology in adoptive immunotherapy is the CRISPR material's delivery vehicle. For example, viral vectors are usually employed to deliver gRNA and Cas9 to mammalian cells. There is a high chance of the immune response triggered by the delivery vehicle or the Cas9 protein. Viral vectors are sometimes known for their immunogenicity, and the Cas9 proteins (considering their microbial origin) could serve as a potential immunogen, thus limiting their use for gene therapy.^132^ Although an increasing number of CRISPR‐Cas9 enzymes have been discovered to date, it is interesting that only two class 2 enzymes (Cas9 and Cas12a) have gained popularity for their use in genome editing.

Also, it is necessary to carefully study and evaluate which of these variants is best suited to the workflow; for example, the different variants of the Cas9 enzymes have individual advantages and disadvantages that should be considered (Table 2). Further extensive research will discover more novel Cas protein variants alongside their unique functionality, which will open up further possibilities in genome engineering.

**Table 2:** Variants of the Cas9 (type II) enzyme of the CRISPR system

Variant	Attributes	Reaction	Advantages
CRISPR‐Cas9 WT	Cas9, sgRNA	Induces double‐strand break at the target site	Highly versatile, stable, easy accessibility and effective
CRISPRa	dCas9, presence of activator peptide, sgRNA	Increase transcription	Has low toxicity
CRISPRi	dCas9, has a repressor peptide, sgRNA	Capable of blocking transcription elongation or knockdown of transcripts	Can be inducible, reversible, possesses low off‐target effects
CRISPR‐Cas9 Nickase	Mutant Cas9 H840A or D10A, sgRNA	Induces a single‐strand break	Convenient, highly robust, efficient, flexible, precise, can be scalable

It is pertinent to develop a safe and efficient delivery system for the generally acceptable *in vivo* application of CRISPR‐Cas9 because the insertion of mutagenesis could arise from the vector itself. Although the AAV‐based vectors are currently the preferred mode of delivery on somatic cells, they can infect dividing and non‐dividing cells, evoking a slight immune response.^133^ One of the significant restrictions of the AAVs is their limited cargo capacity with restricted tissue tropism.

Other physical, non‐viral methods (such as microinjection, electroporation) may be used to overcome these hurdles by introducing Cas9‐encoding plasmids, Cas9‐mRNA or a mixture of Cas9 protein and sgRNA directly into the immune cells and tissues of animals. For example, the use of electroporation to directly deliver CRISPR material to CD4^+^ T cells, CD34^+^ stem cells, cancer cells and embryonic stem cells has been shown.^134^, ^135^ Also, the direct delivery of Cas9–sgRNA ribonucleoproteins (RNPs) to the cell via a lipid complex or transfection may also be used. The RNP delivery system offers some advantages compared to viral or non‐viral approaches because it is delivered pre‐assembled with a fast action when it complexes with target DNA. Its Cas9 nuclease also has a shorter duration, which may reduce off‐target effects and increase its efficiency. Other delivery methods, such as hydrodynamic injections, have been highlighted. The introduction of Cas9 containing vectors through the tail vein of adult rodents for successful mutation and gene correction^23^ shows other ways for the direct *in vivo* delivery of the CRISPR system for genetic manipulation.

However, the CRISPR‐Cas9 system's off‐target effects are still a major concern, particularly for CAR‐T therapies. One smart way to protect normal tissues from tumor‐specific T cells is by employing dual receptor circuits termed as the NOT and/or AND gates. In this approach, one CAR receptor targeted at tumor antigen and initialises the kill switch upon encounter with tumor cells can be engineered onto T cells. In this approach, one CAR receptor‐targeted at tumor antigen that initiates the kill switch upon encounter with tumor cells and another inhibitory CAR molecule that expresses the inhibitory signal (such as CTLA‐4 or PD‐1) when in contact with antigens on normal tissue should be engineered onto T cells.^136^ Similarly, another independent research has shown that it is possible upon the recognition of one antigen to drive the transcription of a CAR specific for a second antigen, allowing for a more‐targeted CAR expression with accompanying reduced off‐target toxicities.^137^ For this approach, the CRISPR‐Cas9 system can simultaneously express the ‘NOT and/or AND gates’ CAR receptor, particularly in overcoming antigens expressed on both normal and tumor tissues. Although this approach sounds exciting, there is a need for an extensive preclinical study to optimise CAR combination that fits well for maximum tumor impact.

The CRISPR genome‐wide screen's concern is the conditional false‐positive generated during the dropout screenings in cancers with aneuploidy. Also, the excessive DSBs – encountered in gene regions with multiple copy numbers, including those of non‐expressed genes – can often result in DNA damage and ultimately apoptosis; therefore, excluding sgRNAs targeting non‐expressed genes from the libraries will avert this. Lastly, since conventionally, sgRNAs are designed to target the 5’ exon, false‐negative results arising from initiation points of genes from other exons implying that the position of sgRNA is critical to the accuracy of the screening outcomes.^138^

Other concerns include the risks of neurological toxicity and cytokine release syndrome whenever CRISPR‐Cas9 is used for any adoptive immunotherapy transfer (including CAR‐T and CAR‐NK). It is imperative to have clinicians who are well trained to manage any unintended adverse effects that may ensue. Another approach is to ensure a comprehensive and thorough study of the safety of these cell‐based therapeutic, particularly at the preclinical level. This will allow the opportunity to evaluate the safety and efficacy of these cell‐based therapies before human studies. It will also uncover unintended safety issues usually revealed in early‐stage clinical trials.

The CRISPR system has revolutionised and championed novel ways of managing haematologic malignancies via CAR‐T and CAR‐NK. There remain many obstacles to broaden its application on solid tumors. The possibilities that can be achieved with the CRISPR system are endless. With the current advances made in immune cell gene editing, T and NK cell engineering, as well as optimised cell manufacturing protocols, have the potential to broaden T and NK cell‐based therapies to other cell types such as hematopoietic stem cells, induced pluripotent stem cells, including macrophages – which recently entered immunotherapy for treating solid tumors^139^, ^140^ – to foster the development of new cell‐based therapies that are beyond oncology into other areas such as organ transplantation, infectious diseases and autoimmunity.

### Overcoming challenges of immune suppression

To optimise immunotherapy regimes for complete tumor regression, the stimulation of robust antitumor response is required. However, overcoming the plethora of immunosuppressive mechanisms, particularly within the TME, remains a challenge. The use of CRISPR‐Cas9 to develop highly effective tumor‐infiltrating lymphocytes capable of penetrating the microenvironment and overcome the suppressive effects of immunosuppressive agents (such as cytokines and growth factors) synthesised by the tumor or stromal cells is desirable.

Transforming growth factor‐beta (TGF‐β) represents one of the pleiotropic immunosuppressive cytokines shown to inhibit T‐cell proliferation, activation and differentiation^141^; similarly, its suppressive role on NK cells has also been well described.^142^, ^143^ In addition, its elevated serum level is often associated as a poor prognosis marker in several malignancies.^144^ TGF‐β has since been shown to exert immunosuppressive activity on cytotoxic lymphocytes by suppressing the expression of cytolytic products such as granzyme A and B, perforin, IFN‐γ and FasL.

Therefore, approaches focused on using the CRISPR system to impair TGF‐β signalling on immune effector cells will significantly enhance their antitumor capabilities.^145^ Additionally, coadministration of anti‐TGFβR2 monoclonal antibody together with small molecule drugs that disrupt TGF‐β‐mediated Smad 3 and 4 signalling is desirous.^146^ By controlling the signalling axis of the various immune checkpoints with mAb or gene knockout using the CRISPR system, offers a vital strategy to overcome the immune‐suppressive environment. Since Treg produces a high amount of TGF,^147^, ^148^ approaches such as endogenous knockout of TGF‐β receptor II (TGFBR2) with CRISPR/Cas9 have been shown to significantly improve the efficacy of CAR‐T cells and diminish the conversion of Treg;^149^ hence, approaches that disrupt the suppressive effect of these regulatory cells including MDSCs will offer unprecedented success.

The presence of other cytokines, including IL‐10, sialomucins and prostaglandin E2, which have been shown to protect tumor cells against T‐cell cytotoxicity, should be investigated. Finally, knocking out diacylglycerol kinase (DGKζ) – an enzyme that converts diacylglycerol to phosphatidic acid – with CRISPR/Cas9 enhances CD3 signalling bolstering TCR signalling and T‐cell functions.^150^ Similarly, knockout of DGKζ has been shown to improve cytokine production, degranulation and effector function of NK cells.^151^

In addition to overcoming the immunosuppressive agents associated with the TME, the CRISPR/Cas9 system has also been used as a novel strategy to study the TME and device new treatment options in transgenic mice, offering the direct capability to induce specific genetic modifications in any working genetic background.^152^ Therefore, employing the CRISPR system's multiplex advantages will offer the opportunity to create highly effective, next‐generation T‐ and NK‐cell CARs to improve immunotherapy.

The numerous immunosuppressive factors found at the tumor site must be overcome to successfully apply CAR‐T and CAR‐NK in solid tumors. Combination strategies such as immune checkpoint and CAR molecules have been reported to yield positive results in this regard.^153^ Another approach is to incorporate additional transgenes so that CAR‐T cells can secrete PD‐1 blocking scFv or anti‐PD‐L1 antibodies at the tumor site simultaneously, enabling the full antitumor function of these tumor‐infiltrating super CAR‐T cells and other intratumoral T cells. In a similar vein, synthetic Notch ‘synNotch’ receptors have been implicated in driving both PD‐1 and CTLA‐4 inhibitors production.^154^ Hence, the inclusion of fusion receptors such as interleukin (IL)‐4–IL‐7 chimeric cytokine receptors has the propensity to shift the inhibitory signals from IL‐4 to IL‐7 signalling – leading to proliferation and memory differentiation of T cells at the tumor site.^155^ To achieve all the above‐described innovative immunotherapy approaches, the CRISPR‐Cas9 technology will be of immense benefit since its multiplex ability allows for the simultaneous knock‐in and knockout of genes *in vitro* and *in vivo*. The future of personalised and highly sophisticated immune therapies may lie in fully exploiting this technology.

Besides identifying the mechanisms that regulate PD‐L1 expression, other approaches contributing to immune evasion and acquired resistance to ICB, such as low MHC class I expression,^156^, ^157^ hold many potential. In a recent study, the genome‐wide CRISPR screen was applied in K562 tumor cells (known for their low MHC‐I expression) and cancer cell lines in which an evolutionarily conserved polycomb repressive complex 2 (PRC2) protein was identified and implicated in the transcriptional regulation of MHC‐I antigen processing pathway (MHC‐I APP), which highlights the tight epigenetic control of MHC‐I expression in these tumor cells. This approach can explore the mechanisms that facilitate increased MHC‐I levels for antigen presentation‐licensing cytotoxic lymphocytes to kill tumor cells.^127^ Other immune exhaustion markers such as CD39 and TOX, as well as those recently been identified (e.g. TIGIT, TIM‐3, CTLA‐4) and their respective ligands in tumors, can be screened to identify their regulation and how their expression pattern can be modulated to improve tumor‐infiltrating lymphocytes activation in combination with ICB therapies.

Another major challenge for cancer immunotherapies is tumor relapse brought about by pre‐existing heterogeneity or downregulation of target antigens reported in CD19^+^ B‐cell‐derived malignancies such as acute lymphoblastic leukaemia.^158^, ^159^, ^160^ To deal with this tumor escape arising from a single‐antigen target, a pan‐cancer antigen can be employed. It involves approaches such as the integration of multiple autonomous CARs using a single vector (e.g. bicistronic CAR),^161^ coadministration of separately transduced CAR‐T cells,^162^ integration of two CARs to a single molecule (tandem CAR)^163^ and co‐transduction of multi‐CAR vector on T cells are currently being tested.

Since T and NK cells are prone to exhaustion at tumor sites, switching their receptor extracellular domain using the CRISPR‐Cas9 system can salvage this phenomenon. For example, fusing the extracellular PD‐1 domain to an intracellular CD28 domain led to activated CAR‐T being less susceptible to exhaustion with an enhanced *in vivo* antitumor activity.^164^ CRISPR‐Cas9 technology was also used to completely overcome the suppressive signalling from PD‐1 through its deletion in CAR‐T before its infusion.^165^ Other CRISPR‐Cas9 system‐mediated clinical trial targeted towards melanoma, synovial sarcoma or MM is underway. TCR mispairing is also restricted by deleting endogenous TCR and PD‐1 with a vector encoding the NY‐ESO‐1‐specific HLA‐A2.^166^

Other laudable approaches include using CAR‐T cells capable of secreting cytokines such as IL‐12,^167^ or those with herpesvirus entry mediator,^168^ and nanoparticles with adenosine receptor antagonists^169^ or a IL‐15 super‐agonist^170^ have all been shown to have potential to revolutionise the next‐generation CAR molecules. Finally, synNotch receptors can deliver cytokines and bispecific antibodies to the tumors.^154^ These innovative approaches offer the avenue to modulate the local TME while augmenting CAR‐based therapies devoid of host systemic effects.

Although we are still far from harnessing the full potential of CRISPR‐based technology, giant strides have been made in genomic research, gene editing and immune cell therapy. Many scientists can now manipulate biological samples (both *in vitro* and *in vivo*) to gain more insights, test hypotheses and answer fundamental scientific questions through the CRISPR technique. Clinicians are also expected to have more robust diagnostic and treatment options, as the much talked about personalised and precision medicine has been brought to the limelight through CRISPR‐based technology. Since CRISPR‐Cas9 has somewhat become the golden standard technology in genetic and biomolecular engineering, it is evident that unlocking the full capability of this technology for cancer research and therapy will improve lives.

## Conflicts of interest

The authors declare no competing interests.

## Author contributions

**Lukman Olalekan Afolabi:** Conceptualization; Writing‐original draft; Writing‐review & editing. **Mariam Olanrewaju Afolabi:** Conceptualization; Writing‐original draft; Writing‐review & editing. **Musbahu Muhammad Sani:** Conceptualization; Writing‐original draft; Writing‐review & editing. **Wahab Oluwanisola Okunowo:** Conceptualization; Writing‐original draft; Writing‐review & editing. **Liang Chen:** Validation; Writing‐review & editing. **Dehong Yan:** Validation; Writing‐review & editing. **Yaou Zhang:** Supervision; Validation; Writing‐review & editing. **Xiaochun Wan:** Funding acquisition; Supervision; Writing‐review & editing.

## Funding

This work was funded by the National Key R&D Program of China (2019YFA0906100), Key‐Area Research and Development Program of Guangdong Province (2019B020201014).

## References

[1] Cooper GM . The Cell: A Molecular Approach. 2nd edition. Sunderland (MA): Sinauer Associates; 2000. The Development and Causes of Cancer. *NCBI Bookshelf* 2000. Available from: https://www.ncbi.nlm.nih.gov/books/NBK9963/

[2] Batool A , Malik F , Andrabi KI . Expansion of the CRISPR/Cas genome‐sculpting toolbox: innovations, applications and challenges. Mol Diagn Ther 2021; 25: 41–57. 33185860 10.1007/s40291-020-00500-8

[3] Zhang B . CRISPR/Cas gene therapy. J Cell Physiol 2021; 236: 2459–2481. 32959897 10.1002/jcp.30064

[4] Naeem M , Majeed S , Hoque MZ *et al*. Latest developed strategies to minimize the off‐target effects in CRISPR‐Cas‐mediated genome editing. Cells 2020; 9: 1608. 10.3390/cells9071608 PMC7407193 32630835

[5] Gruzdev A , Scott GJ , Hagler TB *et al*. CRISPR/Cas9‐assisted genome editing in murine embryonic stem cells. Methods Mol Biol 2019; 1960: 1–21. 30798517 10.1007/978-1-4939-9167-9_1

[6] Singh V , Gohil N , García RR *et al*. Recent advances of CRISPR‐Cas9 genome editing technologies for biological and biomedical investigations. J Cell Biochem 2018; 119: 81–94. 28544016 10.1002/jcb.26165

[7] Zhang D , Hussain A , Manghwar H *et al*. Genome editing with the CRISPR‐Cas system: an art, ethics and global regulatory perspective. Plant Biotechnol J 2020; 18: 1651–1669. 32271968 10.1111/pbi.13383 PMC7336378

[8] Barrangou R , Fremaux C , Deveau H *et al*. CRISPR provides acquired resistance against viruses in prokaryotes. Science 2007; 315: 1709–1712. 17379808 10.1126/science.1138140

[9] Lin J , Zhou Y , Liu J *et al*. Progress and application of CRISPR/Cas technology in biological and biomedical investigation. J Cell Biochem 2017; 118: 3061–3071. 28590031 10.1002/jcb.26198

[10] Xu Y , Li Z . CRISPR‐Cas systems: Overview, innovations and applications in human disease research and gene therapy. Comput Struct Biotechnol J 2020; 18: 2401–2415. 33005303 10.1016/j.csbj.2020.08.031 PMC7508700

[11] Bolotin A , Quinquis B , Sorokin A *et al*. Clustered regularly interspaced short palindrome repeats (CRISPRs) have spacers of extrachromosomal origin. Microbiology 2005; 151: 2551–2561. 16079334 10.1099/mic.0.28048-0

[12] Nuñez JK , Harrington LB , Doudna JA . Chemical and biophysical modulation of Cas9 for tunable genome engineering. ACS Chem Biol 2016; 11: 681–688. 26857072 10.1021/acschembio.5b01019

[13] Hsu PD , Lander ES , Zhang F . Development and applications of CRISPR‐Cas9 for genome engineering. Cell 2014; 157: 1262–1278. 24906146 10.1016/j.cell.2014.05.010 PMC4343198

[14] Cao J , Wu L , Zhang S‐M *et al*. An easy and efficient inducible CRISPR/Cas9 platform with improved specificity for multiple gene targeting. Nucleic Acids Res 2016; 44: e149. 27458201 10.1093/nar/gkw660 PMC5100567

[15] Jinek M , Chylinski K , Fonfara I *et al*. A programmable dual‐RNA–guided DNA endonuclease in adaptive bacterial immunity. Science 2012; 337: 816–821. 22745249 10.1126/science.1225829 PMC6286148

[16] Miyaoka Y , Berman JR , Cooper SB *et al*. Systematic quantification of HDR and NHEJ reveals effects of locus, nuclease, and cell type on genome‐editing. Sci Rep 2016; 6: 23549. 27030102 10.1038/srep23549 PMC4814844

[17] Ray U , Raghavan SC . Modulation of DNA double‐strand break repair as a strategy to improve precise genome editing. Oncogene 2020; 39: 6393–6405. 32884115 10.1038/s41388-020-01445-2

[18] Gratz SJ , Ukken FP , Rubinstein CD *et al*. Highly specific and efficient CRISPR/Cas9‐catalyzed homology‐directed repair in Drosophila. Genetics 2014; 196: 961–971. 24478335 10.1534/genetics.113.160713 PMC3982687

[19] McVey M , Lee SE . MMEJ repair of double‐strand breaks (director’s cut): deleted sequences and alternative endings. Trends Genet 2008; 24: 529–538. 18809224 10.1016/j.tig.2008.08.007 PMC5303623

[20] Sakuma T , Nakade S , Sakane Y *et al*. MMEJ‐assisted gene knock‐in using TALENs and CRISPR‐Cas9 with the PITCh systems. Nat Protoc 2016; 11: 118–133. 26678082 10.1038/nprot.2015.140

[21] Gratz SJ , Cummings AM , Nguyen JN *et al*. Genome engineering of Drosophila with the CRISPR RNA‐guided Cas9 nuclease. Genetics 2013; 194: 1029–1035. 23709638 10.1534/genetics.113.152710 PMC3730909

[22] Wang H , Yang H , Shivalila CS *et al*. One‐step generation of mice carrying mutations in multiple genes by CRISPR/Cas‐mediated genome engineering. Cell 2013; 153: 910–918. 23643243 10.1016/j.cell.2013.04.025 PMC3969854

[23] Xue W , Chen S , Yin H *et al*. CRISPR‐mediated direct mutation of cancer genes in the mouse liver. Nature 2014; 514: 380. 25119044 10.1038/nature13589 PMC4199937

[24] Platt RJ , Chen S , Zhou Y *et al*. CRISPR‐Cas9 knockin mice for genome editing and cancer modeling. Cell 2014; 159: 440–455. 25263330 10.1016/j.cell.2014.09.014 PMC4265475

[25] Sachdeva M , Sachdeva N , Pal M *et al*. CRISPR/Cas9: molecular tool for gene therapy to target genome and epigenome in the treatment of lung cancer. Cancer Gene Ther 2015; 22: 509. 26494554 10.1038/cgt.2015.54

[26] Li J , Shou J , Guo Y *et al*. Efficient inversions and duplications of mammalian regulatory DNA elements and gene clusters by CRISPR/Cas9. J Mol Cell Biol 2015; 7: 284–298. 25757625 10.1093/jmcb/mjv016 PMC4524425

[27] Maddalo D , Manchado E , Concepcion CP *et al*. In vivo engineering of oncogenic chromosomal rearrangements with the CRISPR/Cas9 system. Nature 2014; 516: 423. 25337876 10.1038/nature13902 PMC4270925

[28] Liu Y , Zeng Y , Liu L *et al*. Synthesizing AND gate genetic circuits based on CRISPR‐Cas9 for identification of bladder cancer cells. Nat Commun 2014; 5: 5393. 25373919 10.1038/ncomms6393

[29] Kasap C , Elemento O , Kapoor TM . DrugTargetSeqR: a genomics‐and CRISPR‐Cas9–based method to analyze drug targets. Nat Chem Biol 2014; 10: 626. 24929528 10.1038/nchembio.1551 PMC4123312

[30] Neggers JE , Vercruysse T , Jacquemyn M *et al*. Identifying drug‐target selectivity of small‐molecule CRM1/XPO1 inhibitors by CRISPR/Cas9 genome editing. Chem Biol 2015; 22: 107–116. 25579209 10.1016/j.chembiol.2014.11.015

[31] Shi J , Wang E , Milazzo JP *et al*. Discovery of cancer drug targets by CRISPR‐Cas9 screening of protein domains. Nat Biotechnol 2015; 33: 661–667. 25961408 10.1038/nbt.3235 PMC4529991

[32] Zhen S , Hua L , Takahashi Y *et al*. *In vitro* and *in vivo* growth suppression of human papillomavirus 16‐positive cervical cancer cells by CRISPR/Cas9. Biochem Biophys Res Commun 2014; 450: 1422–1426. 25044113 10.1016/j.bbrc.2014.07.014

[33] Konermann S , Brigham MD , Trevino AE *et al*. Genome‐scale transcriptional activation by an engineered CRISPR‐Cas9 complex. Nature 2015; 517: 583–588. 25494202 10.1038/nature14136 PMC4420636

[34] Gilbert LA , Larson MH , Morsut L *et al*. CRISPR‐mediated modular RNA‐guided regulation of transcription in eukaryotes. Cell 2013; 154: 442–451. 23849981 10.1016/j.cell.2013.06.044 PMC3770145

[35] Koike‐Yusa H , Li Y , Tan EP *et al*. Genome‐wide recessive genetic screening in mammalian cells with a lentiviral CRISPR‐guide RNA library. Nat Biotechnol 2014; 32: 267–273. 24535568 10.1038/nbt.2800

[36] Zhou Y , Zhu S , Cai C *et al*. High‐throughput screening of a CRISPR/Cas9 library for functional genomics in human cells. Nature 2014; 509: 487–491. 24717434 10.1038/nature13166

[37] Sun W , He B , Yang B *et al*. Genome‐wide CRISPR screen reveals SGOL1 as a druggable target of sorafenib‐treated hepatocellular carcinoma. Lab Invest 2018; 98: 734–744. 29467456 10.1038/s41374-018-0027-6

[38] Ouyang Q , Liu Y , Tan J *et al*. Loss of ZNF587B and SULF1 contributed to cisplatin resistance in ovarian cancer cell lines based on Genome‐scale CRISPR/Cas9 screening. Am J Cancer Res 2019; 9: 988–998. 31218106 PMC6556596

[39] Yang B , Kuang J , Wu C *et al*. Screening genes promoting exit from naive pluripotency based on genome‐scale CRISPR‐Cas9 knockout. Stem Cells Int 2020; 2020: 8483035. 32089710 10.1155/2020/8483035 PMC7023212

[40] Du X , Shen X , Dai L *et al*. PSMD12 promotes breast cancer growth via inhibiting the expression of pro‐apoptotic genes. Biochem Biophys Res Commun 2020; 526: 368–374. 32222279 10.1016/j.bbrc.2020.03.095

[41] Zhou X , Li R , Jing R *et al*. Genome‐wide CRISPR knockout screens identify ADAMTSL3 and PTEN genes as suppressors of HCC proliferation and metastasis, respectively. J Cancer Res Clin Oncol 2020; 146: 1509–1521. 32266537 10.1007/s00432-020-03207-9 PMC11804363

[42] Tzelepis K , Koike‐Yusa H , De Braekeleer E *et al*. A CRISPR dropout screen identifies genetic vulnerabilities and therapeutic targets in acute myeloid leukemia. Cell Rep 2016; 17: 1193–1205. 27760321 10.1016/j.celrep.2016.09.079 PMC5081405

[43] Shi CX , Kortüm KM , Zhu YX *et al*. CRISPR genome‐wide screening identifies dependence on the proteasome subunit PSMC6 for bortezomib sensitivity in multiple myeloma. Mol Cancer Ther 2017; 16: 2862–2870. 28958990 10.1158/1535-7163.MCT-17-0130 PMC5796678

[44] Manguso RT , Pope HW , Zimmer MD *et al*. In vivo CRISPR screening identifies Ptpn2 as a cancer immunotherapy target. Nature 2017; 547: 413–418. 28723893 10.1038/nature23270 PMC5924693

[45] Zhang JP , Song Z , Wang HB *et al*. A novel model of controlling PD‐L1 expression in ALK^+^ anaplastic large cell lymphoma revealed by CRISPR screening. Blood 2019; 134: 171–185. 31151983 10.1182/blood.2019001043 PMC6624970

[46] Liu J , Song T , Zhou W *et al*. A genome‐scale CRISPR‐Cas9 screening in myeloma cells identifies regulators of immunomodulatory drug sensitivity. Leukemia 2019; 33: 171–180. 30026574 10.1038/s41375-018-0205-y PMC6475089

[47] Doench JG . Am I ready for CRISPR? A user's guide to genetic screens. Nat Rev Genet 2018; 19: 67–80. 29199283 10.1038/nrg.2017.97

[48] Shifrut E , Carnevale J , Tobin V *et al*. Genome‐wide CRISPR screens in primary human T cells reveal key regulators of immune function. Cell 2018; 175: 1958–1971. 30449619 10.1016/j.cell.2018.10.024 PMC6689405

[49] Singh N , Lee YG , Shestova O *et al*. Impaired death receptor signaling in leukemia causes antigen‐independent resistance by inducing CAR T‐cell dysfunction. Cancer Discov 2020; 10: 552–567. 32001516 10.1158/2159-8290.CD-19-0813 PMC7416790

[50] Dufva O , Koski J , Maliniemi P *et al*. Integrated drug profiling and CRISPR screening identify essential pathways for CAR T‐cell cytotoxicity. Blood 2020; 135: 597–609. 31830245 10.1182/blood.2019002121 PMC7098811

[51] Wang D , Prager BC , Gimple RC *et al*. CRISPR Screening of CAR T Cells and Cancer Stem Cells Reveals Critical Dependencies for Cell‐Based Therapies. Cancer Discov 2020; 11: 1–20. 10.1158/2159-8290.CD-20-1243 PMC8406797 33328215

[52] Roth TL , Li PJ , Blaeschke F *et al*. Pooled Knockin Targeting for Genome Engineering of Cellular Immunotherapies. Cell 2020; 181: 728–744. 32302591 10.1016/j.cell.2020.03.039 PMC7219528

[53] Couzin‐Frankel J . Breakthrough of the year 2013. Cancer immunotherapy. Science 2013; 342: 1432–1433. 24357284 10.1126/science.342.6165.1432

[54] Chevolet I , Speeckaert R , Schreuer M *et al*. Characterization of the in vivo immune network of IDO, tryptophan metabolism, PD‐L1, and CTLA‐4 in circulating immune cells in melanoma. Oncoimmunology 2015; 4: e982382. 25949897 10.4161/2162402X.2014.982382 PMC4404886

[55] June CH , Sadelain M . Chimeric antigen receptor therapy. N Engl J Med 2018; 379: 64–73. 29972754 10.1056/NEJMra1706169 PMC7433347

[56] Cho JH , Collins JJ , Wong WW . Universal chimeric antigen receptors for multiplexed and logical control of T cell responses. Cell 2018; 173: 1426–1438. 29706540 10.1016/j.cell.2018.03.038 PMC5984158

[57] Landgraf KE , Williams SR , Steiger D *et al*. convertible CARs: A chimeric antigen receptor system for flexible control of activity and antigen targeting. Commun Biol 2020; 3: 1–13. 32518350 10.1038/s42003-020-1021-2 PMC7283332

[58] Hu Y , Tian Z‐G , Zhang C . Chimeric antigen receptor (CAR)‐transduced natural killer cells in tumor immunotherapy. Acta Pharmacol Sin 2018; 39: 167–176. 28880014 10.1038/aps.2017.125 PMC5800464

[59] Kochenderfer JN , Rosenberg SA . Treating B‐cell cancer with T cells expressing anti‐CD19 chimeric antigen receptors. Nat Rev Clin Oncol 2013; 10: 267–276. 23546520 10.1038/nrclinonc.2013.46 PMC6322669

[60] Liu X , Zhang Y , Cheng C *et al*. CRISPR‐Cas9‐mediated multiplex gene editing in CAR‐T cells. Cell Res 2017; 27: 154–157. 27910851 10.1038/cr.2016.142 PMC5223227

[61] Torikai H , Reik A , Liu P‐Q *et al*. A foundation for universal T‐cell based immunotherapy: T cells engineered to express a CD19‐specific chimeric‐antigen‐receptor and eliminate expression of endogenous TCR. Blood 2012; 119: 5697–5705. 22535661 10.1182/blood-2012-01-405365 PMC3382929

[62] Riolobos L , Hirata RK , Turtle CJ *et al*. HLA engineering of human pluripotent stem cells. Mol Ther 2013; 21: 1232–1241. 23629003 10.1038/mt.2013.59 PMC3677304

[63] Ren J , Liu X , Fang C *et al*. Multiplex genome editing to generate universal CAR T cells resistant to PD1 inhibition. Clin Cancer Res 2017; 23: 2255–2266. 27815355 10.1158/1078-0432.CCR-16-1300 PMC5413401

[64] Eyquem J , Mansilla‐Soto J , Giavridis T *et al*. Targeting a CAR to the TRAC locus with CRISPR/Cas9 enhances tumour rejection. Nature 2017; 543: 113–117. 28225754 10.1038/nature21405 PMC5558614

[65] Hubbard N , Hagin D , Sommer K *et al*. Targeted gene editing restores regulated CD40L function in X‐linked hyper‐IgM syndrome. Blood 2016; 127: 2513–2522. 26903548 10.1182/blood-2015-11-683235

[66] Lombardo A , Cesana D , Genovese P *et al*. Site‐specific integration and tailoring of cassette design for sustainable gene transfer. Nat Methods 2011; 8: 861–869. 21857672 10.1038/nmeth.1674

[67] Wang J , DeClercq J , Hayward S *et al*. Highly efficient homology‐driven genome editing in human T cells by combining zinc‐finger nuclease mRNA and AAV6 donor delivery. Nucleic Acids Res 2016; 44: e30. 26527725 10.1093/nar/gkv1121 PMC4756813

[68] Afolabi LO , Adeshakin AO , Sani MM *et al*. Genetic reprogramming for NK cell cancer immunotherapy with CRISPR/Cas9. Immunology 2019; 158: 63–69. 31315144 10.1111/imm.13094 PMC6742769

[69] Legut M , Dolton G , Mian AA *et al*. CRISPR‐mediated TCR replacement generates superior anticancer transgenic T cells. Blood 2018; 131: 311–322. 29122757 10.1182/blood-2017-05-787598 PMC5774207

[70] Shao H , Zhang W , Hu Q *et al*. TCR mispairing in genetically modified T cells was detected by fluorescence resonance energy transfer. Mol Biol Rep 2010; 37: 3951–3956. 20373027 10.1007/s11033-010-0053-y

[71] van Loenen MM , de Boer R , Amir AL *et al*. Mixed T cell receptor dimers harbor potentially harmful neoreactivity. Proc Natl Acad Sci USA 2010; 107: 10972–10977. 20534461 10.1073/pnas.1005802107 PMC2890759

[72] Liu X , Zhao Y . CRISPR/Cas9 genome editing: Fueling the revolution in cancer immunotherapy. Curr Res Transl Med 2018; 66: 39–42. 29691200 10.1016/j.retram.2018.04.003

[73] First‐in‐human CRISPR trial. Nat Biotechnol, [updated 2016/08/09; cited 2021/01/10]. Available from: 10.1038/nbt0816-796a.

[74] Stokic‐Trtica V , Diefenbach A , Klose CSN . NK cell development in times of innate lymphoid cell diversity. Front Immunol 2020; 11: 813. 32733432 10.3389/fimmu.2020.00813 PMC7360798

[75] Cózar B , Greppi M , Carpentier S *et al*. Tumor‐infiltrating natural killer cells. Cancer Discov 2020; 11: 34–44. 33277307 10.1158/2159-8290.CD-20-0655 PMC7611243

[76] Bald T , Krummel MF , Smyth MJ *et al*. The NK cell–cancer cycle: advances and new challenges in NK cell–based immunotherapies. Nat Immunol 2020; 21: 835–847. 32690952 10.1038/s41590-020-0728-z PMC8406687

[77] Vivier E , Tomasello E , Baratin M *et al*. Functions of natural killer cells. Nat Immunol 2008; 9: 503–510. 18425107 10.1038/ni1582

[78] Vivier E , Ugolini S , Blaise D *et al*. Targeting natural killer cells and natural killer T cells in cancer. Nat Rev Immunol 2012; 12: 239–252. 22437937 10.1038/nri3174 PMC5161343

[79] Fang F , Xiao W , Tian Z . NK cell‐based immunotherapy for cancer. Semin Immunol 2017; 31: 37–54. 28838796 10.1016/j.smim.2017.07.009

[80] Afolabi L , Bi J , Yang X *et al*. Suppression of the protein quality control system by TRIM30a sensitizes tumor to NK cell immune surveillance. Eur J Immunol 2019; 49: 461–461. 10.1111/imm.13694 37753964

[81] Sivori S , Meazza R , Quintarelli C *et al*. NK Cell‐Based Immunotherapy for Hematological Malignancies. J Clin Med 2019; 8: 1702. 10.3390/jcm8101702 PMC6832127 31623224

[82] Imai C , Mihara K , Andreansky M *et al*. Chimeric receptors with 4–1BB signaling capacity provoke potent cytotoxicity against acute lymphoblastic leukemia. Leukemia 2004; 18: 676–684. 14961035 10.1038/sj.leu.2403302

[83] Liu D , Tian S , Zhang K *et al*. Chimeric antigen receptor (CAR)‐modified natural killer cell‐based immunotherapy and immunological synapse formation in cancer and HIV. Protein Cell 2017; 8: 861–877. 28488245 10.1007/s13238-017-0415-5 PMC5712291

[84] Rezvani K , Rouce RH . The application of natural killer cell immunotherapy for the treatment of cancer. Front Immunol 2015; 6: 578. 26635792 10.3389/fimmu.2015.00578 PMC4648067

[85] Hermanson DL , Kaufman DS . Utilizing chimeric antigen receptors to direct natural killer cell activity. Front Immunol 2015; 6: 195. 25972867 10.3389/fimmu.2015.00195 PMC4412125

[86] Velasquez MP , Szoor A , Bonifant CL *et al*. Two‐pronged cell therapy for B‐cell malignancies: engineering NK cells to target CD22 and redirect bystander T cells to CD19. Blood 2016; 128: 4560.

[87] Mohammed S , Sukumaran S , Bajgain P *et al*. Improving chimeric antigen receptor‐modified T cell function by reversing the immunosuppressive tumor microenvironment of pancreatic cancer. Mol Ther 2017; 25: 249–258. 28129119 10.1016/j.ymthe.2016.10.016 PMC5363304

[88] Li L , Gao Y , Srivastava R *et al*. Lentiviral delivery of combinatorial CAR/CRISPRi circuit into human primary T cells is enhanced by TBK1/IKKɛ complex inhibitor BX795. J Transl Med 2020; 18: 1–12. 32967676 10.1186/s12967-020-02526-2 PMC7510327

[89] Bi J , Tian Z . NK cell exhaustion. Front Immunol 2017; 8: 760. 28702032 10.3389/fimmu.2017.00760 PMC5487399

[90] Han J , Chu J , Keung Chan W *et al*. CAR‐engineered NK cells targeting wild‐type EGFR and EGFRvIII enhance killing of glioblastoma and patient‐derived glioblastoma stem cells. Sci Rep 2015; 5: 11483. 26155832 10.1038/srep11483 PMC4496728

[91] Kruschinski A , Moosmann A , Poschke I *et al*. Engineering antigen‐specific primary human NK cells against HER‐2 positive carcinomas. Proc Natl Acad Sci USA 2008; 105: 17481–17486. 18987320 10.1073/pnas.0804788105 PMC2582261

[92] Chu J , Deng Y , Benson DM *et al*. CS1‐specific chimeric antigen receptor (CAR)‐engineered natural killer cells enhance *in vitro* and *in vi*vo antitumor activity against human multiple myeloma. Leukemia 2014; 28: 917–927. 24067492 10.1038/leu.2013.279 PMC3967004

[93] Raulet DH , Gasser S , Gowen BG *et al*. Regulation of ligands for the NKG2D activating receptor. Annu Rev Immunol 2013; 31: 413–441. 23298206 10.1146/annurev-immunol-032712-095951 PMC4244079

[94] Zhang T , Lemoi BA , Sentman CL . Chimeric NK‐receptor‐bearing T cells mediate antitumor immunotherapy. Blood 2005; 106: 1544–1551. 15890688 10.1182/blood-2004-11-4365 PMC1895219

[95] Chang YH , Connolly J , Shimasaki N *et al*. A chimeric receptor with NKG2D specificity enhances natural killer cell activation and killing of tumor cells. Cancer Res 2013; 73: 1777–1786. 23302231 10.1158/0008-5472.CAN-12-3558

[96] Müller N , Michen S , Tietze S *et al*. Engineering NK cells modified with an EGFRvIII‐specific chimeric antigen receptor to overexpress CXCR4 improves immunotherapy of CXCL12/SDF‐1α‐secreting glioblastoma. J Immunother 2015; 38: 197–210. 25962108 10.1097/CJI.0000000000000082 PMC4428685

[97] Töpfer K , Cartellieri M , Michen S *et al*. DAP12‐based activating chimeric antigen receptor for NK cell tumor immunotherapy. J Immunol 2015; 194: 3201–3212. 25740942 10.4049/jimmunol.1400330

[98] Liu E , Tong Y , Dotti G *et al*. Cord blood NK cells engineered to express IL‐15 and a CD19‐targeted CAR show long‐term persistence and potent antitumor activity. Leukemia 2018; 32: 520–531. 28725044 10.1038/leu.2017.226 PMC6063081

[99] Levin AM , Bates DL , Ring AM *et al*. Exploiting a natural conformational switch to engineer an interleukin‐2 'superkine'. Nature 2012; 484: 529–533. 22446627 10.1038/nature10975 PMC3338870

[100] Lu Z , Bi J , Wan X . Artemisinin sensitizes tumor cells to NK cell‐mediated cytolysis. Biochem Biophys Res Commun 2020; 524: 418–423. 32007276 10.1016/j.bbrc.2020.01.094

[101] Afolabi LO , Bi J , Chen L *et al*. A natural product, Piperlongumine (PL), increases tumor cells sensitivity to NK cell killing. Int Immunopharmacol 2021; 96: 107658. 33887610 10.1016/j.intimp.2021.107658

[102] Sekiba K , Yamagami M , Otsuka M *et al*. Transcriptional activation of the MICA gene with an engineered CRISPR‐Cas9 system. Biochem Biophys Res Commun 2017; 486: 521–525. 28322797 10.1016/j.bbrc.2017.03.076

[103] Villegas FR , Coca S , Villarrubia VG *et al*. Prognostic significance of tumor infiltrating natural killer cells subset CD57 in patients with squamous cell lung cancer. Lung Cancer 2002; 35: 23–28. 11750709 10.1016/s0169-5002(01)00292-6

[104] Peng LS , Zhang JY , Teng YS *et al*. Tumor‐associated monocytes/macrophages impair NK‐cell function via TGFβ1 in human gastric cancer. Cancer Immunol Res 2017; 5: 248–256. 28148545 10.1158/2326-6066.CIR-16-0152

[105] Jin S , Deng Y , Hao JW *et al*. NK cell phenotypic modulation in lung cancer environment. PLoS One 2014; 9: e109976. 25299645 10.1371/journal.pone.0109976 PMC4192363

[106] Kremer V , Ligtenberg MA , Zendehdel R *et al*. Genetic engineering of human NK cells to express CXCR2 improves migration to renal cell carcinoma. J Immunother Cancer 2017; 5: 1–13. 28923105 10.1186/s40425-017-0275-9 PMC5604543

[107] Carlsten M , Levy E , Karambelkar A *et al*. Efficient mRNA‐based genetic engineering of human NK cells with high‐affinity CD16 and CCR7 augments rituximab‐induced ADCC against lymphoma and targets NK cell migration toward the lymph node‐associated chemokine CCL19. Front Immunol 2016; 7: 105. 27047492 10.3389/fimmu.2016.00105 PMC4801851

[108] Levy ER , Carlsten M , Childs RW . mRNA transfection to improve NK cell homing to tumors. Methods Mol Biol 2016; 1441: 231–240. 27177670 10.1007/978-1-4939-3684-7_19

[109] Putz EM , Mayfosh AJ , Kos K *et al*. NK cell heparanase controls tumor invasion and immune surveillance. J Clin Invest 2017; 127: 2777–2788. 28581441 10.1172/JCI92958 PMC5490772

[110] Lanier LL . NK cell recognition. Annu Rev Immunol 2005; 23: 225–274. 15771571 10.1146/annurev.immunol.23.021704.115526

[111] André P , Denis C , Soulas C *et al*. Anti‐NKG2A mAb is a checkpoint inhibitor that promotes anti‐tumor immunity by unleashing both T and NK cells. Cell 2018; 175: 1731–1743. 30503213 10.1016/j.cell.2018.10.014 PMC6292840

[112] Zhang Q , Bi J , Zheng X *et al*. Blockade of the checkpoint receptor TIGIT prevents NK cell exhaustion and elicits potent anti‐tumor immunity. Nat Immunol 2018; 19: 723–732. 29915296 10.1038/s41590-018-0132-0

[113] Blake SJ , Stannard K , Liu J *et al*. Suppression of metastases using a new lymphocyte checkpoint target for cancer immunotherapy. Cancer Discov 2016; 6: 446–459. 26787820 10.1158/2159-8290.CD-15-0944

[114] Narayanan S , Ahl PJ , Au VB *et al*. LAG3 is a Central Regulator of NK Cell Cytokine Production. bioRxiv 2020. 10.1101/2020.01.31.928200.

[115] Cao Y , Wang X , Jin T *et al*. Immune checkpoint molecules in natural killer cells as potential targets for cancer immunotherapy. Signal Transduct Target Ther 2020; 5: 1–19. 33122640 10.1038/s41392-020-00348-8 PMC7596531

[116] Brahmer JR , Lacchetti C , Schneider BJ *et al*. Management of immune‐related adverse events in patients treated with immune checkpoint inhibitor therapy: American society of clinical oncology clinical practice guideline. J Clin Oncol 2018; 36: 1714–1768. 29442540 10.1200/JCO.2017.77.6385 PMC6481621

[117] Ratner L , Waldmann TA , Janakiram M *et al*. Rapid progression of adult T‐cell leukemia‐lymphoma after PD‐1 inhibitor therapy. N Engl J Med 2018; 378: 1947–1948. 29768155 10.1056/NEJMc1803181

[118] Spain L , Diem S , Larkin J . Management of toxicities of immune checkpoint inhibitors. Cancer Treat Rev 2016; 44: 51–60. 26874776 10.1016/j.ctrv.2016.02.001

[119] Daassi D , Mahoney KM , Freeman GJ . The importance of exosomal PDL1 in tumour immune evasion. Nat Rev Immunol 2020; 20: 209–215. 31965064 10.1038/s41577-019-0264-y

[120] Havel JJ , Chowell D , Chan TA . The evolving landscape of biomarkers for checkpoint inhibitor immunotherapy. Nat Rev Cancer 2019; 19: 133–150. 30755690 10.1038/s41568-019-0116-x PMC6705396

[121] Burr ML , Sparbier CE , Chan YC *et al*. CMTM6 maintains the expression of PD‐L1 and regulates anti‐tumour immunity. Nature 2017; 549: 101–105. 28813417 10.1038/nature23643 PMC5706633

[122] Suresh S , Chen B , Zhu J *et al*. eIF5B drives integrated stress response‐dependent translation of PD‐L1 in lung cancer. Nat Cancer 2020; 1: 533–545. 32984844 10.1038/s43018-020-0056-0 PMC7511089

[123] Palmer DC , Webber BR , Patel Y *et al*. Internal checkpoint regulates T cell neoantigen reactivity and susceptibility to PD1 blockade. bioRxiv 2020. 10.1101/2020.09.24.306571. PMC9847506 36007524

[124] Potts MA , McDonald JA , Sutherland KD *et al*. Critical cancer vulnerabilities identified by unbiased CRISPR/Cas9 screens inform on efficient cancer Immunotherapy. Eur J Immunol 2020; 50: 1871–1884. 33202035 10.1002/eji.202048712

[125] Ishizuka JJ , Manguso RT , Cheruiyot CK *et al*. Loss of ADAR1 in tumours overcomes resistance to immune checkpoint blockade. Nature 2019; 565: 43–48. 30559380 10.1038/s41586-018-0768-9 PMC7241251

[126] Deng H , Tan S , Gao X *et al*. Cdk5 knocking out mediated by CRISPR‐Cas9 genome editing for PD‐L1 attenuation and enhanced antitumor immunity. Acta Pharm Sin B 2020; 10: 358–373. 32082979 10.1016/j.apsb.2019.07.004 PMC7016277

[127] Burr ML , Sparbier CE , Chan KL *et al*. An evolutionarily conserved function of polycomb silences the MHC class I antigen presentation pathway and enables immune evasion in cancer. Cancer Cell 2019; 36: 385–401. 31564637 10.1016/j.ccell.2019.08.008 PMC6876280

[128] Peng D , Kryczek I , Nagarsheth N *et al*. Epigenetic silencing of TH1‐type chemokines shapes tumour immunity and immunotherapy. Nature 2015; 527: 249–253. 26503055 10.1038/nature15520 PMC4779053

[129] Adeegbe DO , Liu Y , Lizotte PH *et al*. Synergistic immunostimulatory effects and therapeutic benefit of combined histone deacetylase and bromodomain inhibition in non‐small cell lung cancer. Cancer Discov 2017; 7: 852–867. 28408401 10.1158/2159-8290.CD-16-1020 PMC5540748

[130] Li X , Wang M , Xiang R . Clonal replacement of novel T cells: a new phenomenon in the tumor microenvironment following PD‐1 blockade. Signal Transduct Target Ther 2019; 4: 43. 31666993 10.1038/s41392-019-0077-2 PMC6813357

[131] Li XY , Moesta AK , Xiao C *et al*. Targeting CD39 in cancer reveals an extracellular ATP‐ and inflammasome‐driven tumor immunity. Cancer Discov 2019; 9: 1754–1773. 31699796 10.1158/2159-8290.CD-19-0541 PMC6891207

[132] Bessis N , GarciaCozar F , Boissier M . Immune responses to gene therapy vectors: influence on vector function and effector mechanisms. Gene Ther 2004; 11: S10–S17. 15454952 10.1038/sj.gt.3302364

[133] Dai W‐J , Zhu L‐Y , Yan Z‐Y *et al*. CRISPR‐Cas9 for in vivo gene therapy: promise and hurdles. Mol Therapy Nucl Acids 2016; 5: e349. 10.1038/mtna.2016.58 PMC5023403 28131272

[134] Hou Z , Zhang Y , Propson NE *et al*. Efficient genome engineering in human pluripotent stem cells using Cas9 from Neisseria meningitidis. Proc Natl Acad Sci 2013; 110: 15644–15649. 23940360 10.1073/pnas.1313587110 PMC3785731

[135] Mandal PK , Ferreira LM , Collins R *et al*. Efficient ablation of genes in human hematopoietic stem and effector cells using CRISPR/Cas9. Cell Stem Cell 2014; 15: 643–652. 25517468 10.1016/j.stem.2014.10.004 PMC4269831

[136] Fedorov VD , Themeli M , Sadelain M . PD‐1‐ and CTLA‐4‐based inhibitory chimeric antigen receptors (iCARs) divert off‐target immunotherapy responses. Sci Transl Med 2013; 5: 215ra172. 10.1126/scitranslmed.3006597 PMC4238416 24337479

[137] Roybal KT , Rupp LJ , Morsut L *et al*. Precision tumor recognition by T cells with combinatorial antigen‐sensing circuits. Cell 2016; 164: 770–779. 26830879 10.1016/j.cell.2016.01.011 PMC4752902

[138] Munoz DM , Cassiani PJ , Li L *et al*. CRISPR screens provide a comprehensive assessment of cancer vulnerabilities but generate false‐positive hits for highly amplified genomic regions. Cancer Discov 2016; 6: 900–913. 27260157 10.1158/2159-8290.CD-16-0178

[139] Klichinsky M , Ruella M , Shestova O *et al*. Human chimeric antigen receptor macrophages for cancer immunotherapy. Nat Biotechnol 2020; 38: 947–953. 32361713 10.1038/s41587-020-0462-y PMC7883632

[140] Mukhopadhyay M . Macrophages enter CAR immunotherapy. Nat Methods 2020; 17: 561. 32499619 10.1038/s41592-020-0862-4

[141] Li MO , Wan YY , Sanjabi S *et al*. Transforming growth factor‐β regulation of immune responses. Annu Rev Immunol 2006; 24: 99–146. 16551245 10.1146/annurev.immunol.24.021605.090737

[142] Batlle E , Massagué J . Transforming growth factor‐β signaling in immunity and cancer. Immunity 2019; 50: 924–940. 30995507 10.1016/j.immuni.2019.03.024 PMC7507121

[143] Zaiatz‐Bittencourt V , Finlay DK , Gardiner CM . Canonical TGF‐β signaling pathway represses human NK cell metabolism. J Immunol 2018; 200: 3934–3941. 29720425 10.4049/jimmunol.1701461

[144] Drake CG , Jaffee E , Pardoll DM . Mechanisms of immune evasion by tumors. Adv Immunol 2006; 90: 51–81. 16730261 10.1016/S0065-2776(06)90002-9

[145] Thomas DA , Massagué J . TGF‐β directly targets cytotoxic T cell functions during tumor evasion of immune surveillance. Cancer Cell 2005; 8: 369–380. 16286245 10.1016/j.ccr.2005.10.012

[146] Kaklamani VG , Pasche B . Role of TGF‐β in cancer and the potential for therapy and prevention. Expert Rev Anticancer Ther 2004; 4: 649–661. 15270668 10.1586/14737140.4.4.649

[147] Rosenberg SA . IL‐2: the first effective immunotherapy for human cancer. J Immunol 2014; 192: 5451–5458. 24907378 10.4049/jimmunol.1490019 PMC6293462

[148] Ghiringhelli F , Ménard C , Terme M *et al*. CD4^+^CD25^+^ regulatory T cells inhibit natural killer cell functions in a transforming growth factor‐β‐dependent manner. J Exp Med 2005; 202: 1075–1085. 16230475 10.1084/jem.20051511 PMC2213209

[149] Tang N , Cheng C , Zhang X *et al*. TGF‐β inhibition via CRISPR promotes the long‐term efficacy of CAR T cells against solid tumors. JCI Insight 2020; 5: e133977. 10.1172/jci.insight.133977 PMC7101140 31999649

[150] Jung IY , Kim YY , Yu HS *et al*. CRISPR/Cas9‐mediated knockout of DGK improves antitumor activities of human T cells. Cancer Res 2018; 78: 4692–4703. 29967261 10.1158/0008-5472.CAN-18-0030

[151] Yang E , Singh BK , Paustian AM *et al*. Diacylglycerol kinase ζ Is a target to enhance NK cell function. J Immunol 2016; 197: 934–941. 27342844 10.4049/jimmunol.1600581 PMC4935923

[152] Yamanaka Y . CRISPR/Cas9 genome editing as a strategy to study the tumor microenvironment in transgenic mice. Methods Mol Biol 2016; 1458: 261–271. 27581028 10.1007/978-1-4939-3801-8_19

[153] Chong EA , Melenhorst JJ , Lacey SF *et al*. PD‐1 blockade modulates chimeric antigen receptor (CAR)‐modified T cells: refueling the CAR. Blood 2017; 129: 1039–1041. 28031179 10.1182/blood-2016-09-738245 PMC5391777

[154] Roybal KT , Williams JZ , Morsut L *et al*. Engineering T cells with customized therapeutic response programs using synthetic notch receptors. Cell 2016; 167: 419–432. 27693353 10.1016/j.cell.2016.09.011 PMC5072533

[155] Leen AM , Sukumaran S , Watanabe N *et al*. Reversal of tumor immune inhibition using a chimeric cytokine receptor. Mol Ther 2014; 22: 1211–1220. 24732709 10.1038/mt.2014.47 PMC4048899

[156] Garrido F , Aptsiauri N , Doorduijn EM *et al*. The urgent need to recover MHC class I in cancers for effective immunotherapy. Curr Opin Immunol 2016; 39: 44–51. 26796069 10.1016/j.coi.2015.12.007 PMC5138279

[157] Lee JH , Shklovskaya E , Lim SY *et al*. Transcriptional downregulation of MHC class I and melanoma de‐ differentiation in resistance to PD‐1 inhibition. Nat Commun 2020; 11: 1897. 32312968 10.1038/s41467-020-15726-7 PMC7171183

[158] Maude SL , Laetsch TW , Buechner J *et al*. Tisagenlecleucel in children and young adults with B‐cell lymphoblastic leukemia. N Engl J Med 2018; 378: 439–448. 29385370 10.1056/NEJMoa1709866 PMC5996391

[159] Turtle CJ , Hanafi LA , Berger C *et al*. CD19 CAR‐T cells of defined CD4^+^:CD8^+^ composition in adult B cell ALL patients. J Clin Invest 2016; 126: 2123–2138. 27111235 10.1172/JCI85309 PMC4887159

[160] Maude SL , Frey N , Shaw PA *et al*. Chimeric antigen receptor T cells for sustained remissions in leukemia. N Engl J Med 2014; 371: 1507–1517. 25317870 10.1056/NEJMoa1407222 PMC4267531

[161] Bielamowicz K , Fousek K , Byrd TT *et al*. Trivalent CAR T cells overcome interpatient antigenic variability in glioblastoma. Neuro Oncol 2018; 20: 506–518. 29016929 10.1093/neuonc/nox182 PMC5909636

[162] Huang L , Wang N , Li C *et al*. Sequential infusion of anti‐CD22 and anti‐CD19 chimeric antigen receptor T cells for adult patients with refractory/relapsed B‐cell acute lymphoblastic leukemia. Blood 2017; 130: 846.

[163] Hegde M , Mukherjee M , Grada Z *et al*. Tandem CAR T cells targeting HER2 and IL13Rα2 mitigate tumor antigen escape. J Clin Invest 2016; 126: 3036–3052. 27427982 10.1172/JCI83416 PMC4966331

[164] Liu X , Ranganathan R , Jiang S *et al*. A chimeric switch‐receptor targeting PD1 augments the efficacy of second‐generation CAR T cells in advanced solid tumors. Cancer Res 2016; 76: 1578–1590. 26979791 10.1158/0008-5472.CAN-15-2524 PMC4800826

[165] Rupp LJ , Schumann K , Roybal KT *et al*. CRISPR/Cas9‐mediated PD‐1 disruption enhances anti‐tumor efficacy of human chimeric antigen receptor T cells. Sci Rep 2017; 7: 737. 28389661 10.1038/s41598-017-00462-8 PMC5428439

[166] Stadtmauer EA , Fraietta JA , Davis MM *et al*. CRISPR‐engineered T cells in patients with refractory cancer. Science 2020; 367: eaba7365. 32029687 10.1126/science.aba7365 PMC11249135

[167] Pegram HJ , Lee JC , Hayman EG *et al*. Tumor‐targeted T cells modified to secrete IL‐12 eradicate systemic tumors without need for prior conditioning. Blood 2012; 119: 4133–4141. 22354001 10.1182/blood-2011-12-400044 PMC3359735

[168] Boice M , Salloum D , Mourcin F *et al*. Loss of the HVEM tumor suppressor in lymphoma and restoration by modified CAR‐T cells. Cell 2016; 167: 405–418. 27693350 10.1016/j.cell.2016.08.032 PMC5221752

[169] Siriwon N , Kim YJ , Siegler E *et al*. CAR‐T cells surface‐engineered with drug‐encapsulated nanoparticles can ameliorate intratumoral T‐cell hypofunction. Cancer Immunol Res 2018; 6: 812–824. 29720380 10.1158/2326-6066.CIR-17-0502

[170] Tang L , Zheng Y , Melo MB *et al*. Enhancing T cell therapy through TCR‐signaling‐responsive nanoparticle drug delivery. Nat Biotechnol 2018; 36: 707–716. 29985479 10.1038/nbt.4181 PMC6078803

